# Supplementary material for: The preference signature of the SARS-CoV-2 Nucleocapsid NTD for its 5’-genomic RNA elements
Source: Nat Commun. 2023 Jun 7;14:3331. doi: 10.1038/s41467-023-38882-y (PMC10246530; doi:10.1038/s41467-023-38882-y)
Supplement: Supplementary file 1 — Supplementary Information [file 41467_2023_38882_MOESM1_ESM.pdf]

The preference signature of the SARS-CoV-2 Nucleocapsid NTD

for its 5'-genomic RNA elements

Sophie Marianne Korn[1,2], Karthikeyan Dhamotharan[1,2], Cy M. Jeffries[3], Andreas Schlundt[1,2*]

[1]Institute for Molecular Biosciences and

[2]Center for Biomolecular Magnetic Resonance (BMRZ), Goethe University Frankfurt, Max-von-Laue-Str. 7-9, 60438 Frankfurt/M., Germany.

[3]European Molecular Biology Laboratory (EMBL) Hamburg Site, c/o Deutsches Elektronen-Synchrotron, Notkestr. 85, 22607 Hamburg, Germany.

[*] To whom correspondence should be addressed: schlundt@bio.uni-frankfurt.de

Supplementary Information

This document contains:

- Supplementary Methods
- 2 Supplementary Tables
- 16 Supplementary Figures
- Supplementary References

Supplementary Methods

Experimental temperatures

Exact temperatures are given in Kelvin for each experiment, either in the Methods section or – if required – in the corresponding figure legend. Whenever experiments were carried out without distinct temperature control, we refer to it as room temperature (RT). By our definition – and to our best knowledge – RT spans a range between 22 and 25°C.

NTD construct cloning

An *Escherichia coli* codon-optimized DNA construct (*Eurofins* Genomics) coding for the SCoV-2 N protein NTD was cloned into the pET-21-based vector pET-Trx1a, containing an N-terminal His_6-tag, a Thioredoxin-tag (Trx) and a tobacco etch virus (TEV) cleavage site via Gibson assembly. A 14.9 kDa protein was obtained upon TEV cleavage, containing one artificial N-terminal glycine residue before the start of the native protein sequence at Gly44 in the full-length N protein sequence. NTD point mutants (G60I, G99I, S105I and R107A) were created by site-directed mutagenesis with primers designed using the NEBaseChanger® tool (see Source Data). With those, the NTD wild-type construct was amplified using Q5 DNA polymerase followed by kinase, ligase and DpnI treatment. The plasmids were then transformed into *E. coli* DH5α and individual clones with point mutations of interest were identified by sequencing.

SEC-SAXS and SEC-SAXS-MALS

SEC-SAXS measurements ($I(q)$ vs. q, where q is defined in equation (1) and 2θ is the scattering angle and λ the X-ray wavelength) were performed at 293 K at the EMBL P12 BioSAXS beamline at PETRAIII (DESY synchrotron Hamburg)[1] equipped with a Pilatus 6M 2D photon counting area detector.

$$\text{(1)} \qquad \mathrm{q} = 4\pi\sin\theta/\lambda$$

All samples were mixed from purified components, shock-frozen in liquid nitrogen, delivered to the beam line, defrozen and subjected to SEC-SAXS after extensive centrifugation. Prior testing revealed that shock-freezing and thawing does not negatively influence sample states. Samples were normally delivered 1-2 days before beamtime and locally stored at -80 °C.

90 µL of protein, RNA and protein-RNA complex (RNP) samples were loaded onto a Superdex75 Increase 10/300GL (GE Healthcare) in standard phosphate buffer using an Agilent 1260 Infinity Bio-Inert HPLC system at a flow rate of 0.6-0.7 mL/min. Between 2000-2520 successive 2D SAXS data frames of 0.995 second each were collected from the

continuously flowing eluate spanning one column volume (24 mL) and delivered directly to the beam line (1-mm capillary). All 2D-data underwent azimuthal averaging using standard P12 beam line data reduction protocols[2] and the resulting 1D-scattering profiles analyzed with CHROMIXS[3] (see Supplementary Fig. 8) and additional modules of the ATSAS 3.0 software suite[4]. After the subtraction of appropriate background/buffer scattering contributions from the SEC-peak sample data frames, we used the CHROMIXS-derived R_g values (calculated through the SEC-elution peaks, combined with the similarity/stability of the scattering profiles spanning the peaks) to guide the final scaling and averaging of the SAXS data and produce the final 1D-background subtracted profiles as reported in the text (Fig. 4, Suppl. Fig. 8 and also see Supplementary Table 2). Due to instrumental effects/beam-line fluctuations and parasitic scattering from the beam-stop, as noted for some of the SEC-SAXS runs, the selection of the appropriate buffer-scattering frames used for background subtraction were based on the evaluation of the influence of these fluctuations on $I(q)$ at the very lowest of angles, near the beam-stop and within the Guinier region of the scattering data ($\ln I(q)$ vs. q^2), i.e. in the range $q_{\min}R_g < qR_g < q_{\max}R_g = 1.3$). For all samples, the individual data frames used for final averaging were selected that produced a consistent/stable R_g (+/- 1 Å) through the selected SEC-elution, and where the averaged buffer used for subtraction minimized the accessible $q^2_{\min}$ in the Guinier region while at the same time avoiding systematic negative scattering intensities at higher angles ($q > 0.4$ Å^{-1}; otherwise indicative of the over-subtraction of buffer scattering contributions). Buffer over-subtraction combined with evaluating parasitic scattering effects were also qualitatively assessed via the calculation of scattering-pair distance distribution functions, or $P(r)$ profiles, using GNOM[5] without $P(0)=0$ and/or $P(D_{max})=0$ constraints enabled, whereby the finally selected averaged buffer scattering generated positive unconstrained values of $P(0)$ near 0, and a decay in $P(r)$ that flattened and limited to 0 toward D_{max} (the maximum particle dimension). The final $P(r)$ curves displayed in Fig. 4, are normalized to the forward scattering intensity, $I(0)$, using the BIOXTAS RAW suite[6] and have $P(0)=0$ and $P(D_{max})=0$ reimposed. As a consequence of these analyses, those data frames forming the central SEC elution peak region were selected for final averaging in the free RNA samples, while for the free NTD, we omitted the right tail of the peak suggesting traces of degradation. For the RNPs, where possible, we focused on the analysis of the left half of complex peaks to avoid contamination by non-complexed species, e.g. in case of SL4 as given by close-by eluting free NTD. For the NTD-SL4ext complex we analyzed the right half of the peak as it was found that the left part represents RNPs of inconsistently larger R_g values either indicating mixed RNP stoichiometries or SEC retention affected by alternative SL4ext conformations, e.g. by unfolding of the Ext RNA (see NMR-data in Supplementary Fig. 11 and 16). The final curves for all samples were processed to derive R_g, Porod volumes, $P(r)$ and molecular weight estimates using Bayesian inference[7] where these structural parameters, and

relevant quality of fit assessments, are reported in Supplementary Table 2 as guided by the recommendations of Trewhella et al.[8] We consistently used R_g values from the real space, i.e. via the $P(r)$.

We used the finally processed SEC-SAXS data to create structural models for SL2+3, SL4, Ext, and SL4ext with RNAmasonry[9] via 50 iterative steps using CRYSOL[10] as a model fit procedure. Secondary structure was manually given for the initial step, based on the models as described in Supplementary Fig. 1 and kept unrestrained for folding and 3D-model building. Final models underwent manual protonation using PyMol (Delano Scientific, Schrödinger). The NTD X-ray crystal structure fit to the SAXS data (PBD: 6M3M) was evaluated using CRYSOL. All SAXS data and relevant models are available in the Small Angle Scattering Biological Databank, SASBDB[11].

In addition to the SEC-SAXS analysis, SEC-SAXS-MALS measurements for the apo NTD were carried out at 20 °C in phosphate buffer via mail-in operation on the EMBL P12 BioSAXS beamline at PETRAIII (DESY, Hamburg)[1] (see SEC-SAXS-MALS procedure below).

To exclude effects of buffer-caused radiation damage, all components were additionally SEC-SAXS-investigated in a HEPES buffer system, including the synchronous recording of MALS (SEC-SAXS-MALS). For this, all samples were transported on ice the day before, mixed on site, centrifuged, prior to measurement. To enable parallel SAXS and MALS data acquisition, the run-out from the column was split and passed, in equal amounts, to the SAXS capillary and the UV-Vis (Agilent variable wavelength UV-Vis detector (VWD), Agilent Technologies), MALS (miniDAWN® TREOS®; Wyatt Technology), and refractive index (RI) detectors (Optilab T-rEX; Wyatt Technology), respectively (in detail described elsewhere[12]). To circumvent possible radiation damage-induced aggregation and to confirm the monodisperse nature of the protein and RNAs alone, SEC-SAXS-MALS on individual components was carried out at 25°C in 25 mM HEPES, 75 mM KCl, 2.5 mM $NaNO_3$, pH 7.2. 90 µL of protein/RNA (~300 µM each, supplemented with 2 mM DTT) were loaded onto a Superdex75 Increase 10/300GL (Cytiva) at a flow rate of 0.7 mL/min. SAXS data and processing as described in the main text. The molar masses of the major SEC elution peaks were calculated from the MALS combined with RI data using ASTRA 7 software (Wyatt Technology) where the protein refractive index increment, dn/dc, was set to 0.185 and 0.175 mL/g for the NTD, and RNA samples, respectively. Additional details on samples, data collection and processing procedures are given in the Source Data file.

Note that all SAXS-derived numbers provided in this manuscript and used for downstream analysis are taken from the manual processing and validation of raw data as described here and shown in the Supplementary Table/Source Data Table. Numbers given in the SASBDB,

usually rounded to no digits left, are based on automated scripts, and do not fully comply with manual processing necessarily.

Analytical Size-exclusion chromatography (aSEC)

Analytical SEC runs at 4 °C were performed by loading 100 µg of protein, protein-RNA complex (100 µg protein with 1.2-fold molar excess of RNA) or 33 µg RNA samples onto a Superdex75 Increase 10/300GL equilibrated with NTD buffer using a Bio-Rad NGC FPLC. Flow rates were set to 0.75 mL/min. The run was monitored with two UV absorbance (260 and 280 nm) traces. All SEC runs were analyzed using ChromLab_v6 (Bio-Rad) and the traces plotted in OriginPro. Quantitative analysis of complex formation between NTD and RNAs at 4 °C and room temperature (RT) was performed as follows: Peak areas of obvious (fully shifted to higher MW and/or providing a separate peak) or expected complexes (as hidden/overlaid with free RNA and or NTD) were determined by integration of the A_{280} curve using the implemented tool in ChromLab. The peak area of free NTD was subtracted in both temperatures in case of overlap, likewise taking into account residual free RNA at the respective retention volume. The ratio between integrals at RT over 4°C was corrected by the A_{260}/A_{280} ratio of the respective free RNA to account for the different specifics of the HPLC UV-lamps and as linewidths of all species' fractions visibly differed between temperatures. By doing so, we also corrected the A_{260}/A_{280} ratios as exemplarily given in Supplementary Fig. 15d (see also Source Data). Altogether, this correction yielded comparable and normalized integrals of RNPs. For RNAs with no peak shifts to higher MW (no lower retention volumes) we compared A_{260}/A_{280} ratios of RNP mixes and RNAs within one RNA and one temperature to verify pure-RNA peaks and position and presence of protein fractions, both together indicating no binding (SL4/NTD-SL4 in Fig. 2). The graphical output was normalized to 280-nm absorption of free NTD for the two temperatures.

aSEC-based MW determination of the NTD

Retention volumes of the Superdex75 Increase 10/300GL column were calibrated at room temperature (RT) by loading 250 µL of premixed commercial low molecular weight standards (Cytiva) containing a mixture of five proteins in NTD standard buffer and running them at 0.75 mL/min. A standard calibration curve was generated from a linear fit of the partition coefficient (K_{av}) versus the log molecular weight of the protein standards. The apparent molecular weight of NTD at two different concentrations (70 µM and 500 µM) was calculated by interpolation from their K_{av} using the equation derived from the linear fit (see Supplementary Fig. 8 and Source Data).

NMR

Due to buffer inconsistencies and ambiguous chemical shifts compared to conditions published by[13,14] we re-confirmed the NTD backbone NMR resonances through an unbiased, independent assignment procedure. To this end we recorded HNCACB, HNCO and HNcaCO spectra[15,16] of uniformly labelled ^{13}C, ^{15}N NTD of 500 µM. Assignments of the NTD were performed using the CCPNMR analysis 2.4 software suite[17] and the program Sparky[18]. All NMR-related effects were mapped and visualized on the NTD using the NMR structure of PDB entry 6YI3[13].

Assignments of imino resonances for Ext RNA were determined with a sample of 300 µM at 278K by means of an imino-proton 2D-NOESY with frequency shift at 600 MHz field strength (220 scans, 4k x 140 points). Given the symmetry of base-pairing in the Ext stemloop, we exploited a U-to-A loop mutant to assign resonances to the upper and lower half of the stem, respectively (see Supplementary Fig. 14). Guided by the available assignments for SL4[19,20], the SL4ext assignment was carried out with a sample of 600 µM at 278 K and 298 K using 2D-imino NOESYs and ^{15}N BEST-TROSYs (nitrogen offset of 153 ppm and spectral width of 25 ppm), and including a high-resolution temperature titration on the imino proton level at 950 MHz field strength. NOESYs were recorded with 150 and 250 ms mixing times at 278 K (2k x 160 points) and 150 ms at 298K (2k x 128 points), each of which with 512 scans. Assignments of Ext imino protons and SL4ext imino groups have deposited in the BMRB under entry IDs 51995 and 51996, respectively. Re-assignment of imino resonances for the SL2+3 RNA was carried out with a sample of 300 µM at 800 MHz field strength and 278 K (1k scans, 2k x 182 points), while the assignment of SL2-derived peaks at 298 K was accomplished based on previous data[20] and a temperature series. All spectral widths were selected based on 1D spectra.

Isothermal titration calorimetry (ITC)

ITC measurements of NTD with SL4, SL4ext and SL1_ext^{SL1} were performed on a VP-ITC200 device (Malvern, United Kingdom) in the standard phosphate NTD buffer, with either 50 mM (low salt) or 250 mM (high salt) KCl, to allow for/abolish contribution of electrostatic interactions. In all replicate experiments, protein was titrated from a stock of 500 µM to 37.5 µM of RNA provided in the reaction cell. We used 19 injections of NTD with 180 s of spacing at 298 K and a stirring speed of 310 rpm. Raw data were analyzed with the NITPIC and SEDPHAT software tools[21]. Heat production was fitted using an unbiased approach testing possible models and finally led to a two-step sequential binding model for all RNAs for comparison. However, at low salt conditions only a single interpretable binding site was fittable for RNAs with the exception of SL4ext (two sited). At high KCl concentration no binding was fittable for RNAs, with the exception of SL4ext (see Source Data file and Supplementary Fig. 4).

For the particular comparison of NTD-SL4 and NTD-SL4ext we also included ITC titrations of RNA added to NTD in low salt conditions. Here, in all replicate experiments, RNA was titrated from a stock of either 500 or 400 µM to 37.5 or 50 µM of NTD provided in the reaction cell. In individual runs, we used either 19 or 39 injections of RNA with 120 to 180 s of spacing adjusted to the optimum resolution of expected transitions at 298 K and a stirring speed of 500 rpm. Heat production was fitted as before and revealed basically identical outcomes for the two complex formations as given above for the inverse titration. For the particularly interesting comparison of NTD binding to SL4 or SL4ext we finally integrated all ITC data measured al low salt condition leading to the distinctive one-site binding (SL4) and two-site binding (SL4ext) reactions with respective affinities shown in Supplementary Fig. 4d.

For all interpretable binding events the fits revealed the respective molar ratios (n), as well as molar Gibbs energies (∆G), binding enthalpies (∆H) and entropies (-T∆S). Baseline-corrected raw data and fitted curves of heat production as a function of molar ratio/concentration were plotted with GUSSI[21]. For all runs, we tested the effect of and, where appropriate, performed data subtraction using NTD titrated to buffer instead of RNA as well as buffer (instead of NTD) titrated to SL4-related RNAs as shown in the Source Data file.

Supplementary Table 1, related to Table 1:

Supplementary Table 1: Overview of RNAs and sequences used in this study. Small letters indicate non-genomic nucleotides artificially added to facilitate T7 *in vitro* transcription.

RNA	Genomic position	nts	Sequence 5’-3’
SL1 [a]	7-33	27	GGUUUAUACCUUCCCAGGUAACAAACC
SL2+3 [a]	40-80	43	ggCUUUCGAUCUCUUGUAGAUCUGUUCUCUAAACGAACUUUAA
SL4 [a]	86-125	44	ggGUGUGGCUGUCACUCGGCUGCAUGCUUAGUGCACUCACGCcc
SL4ext [a] (SL4_ext^{SL4})	83-149	69	ggUCUGUGUGGCUGUCACUCGGCUGCAUGCUUAGUGCACUCACGCAG UAUAAUUAAUAACUAAUUACUG
Ext [a]	129-148	22	ggAUAAUUAAUAACUAAUUACU
Ext_C-A	129-148	22	ggAUAAUUAAUAA**a**UAAUUACU
SL5 [a/b]	149-297	150	gGUCGUUGACAGGACACGAGUAACUCGUCUAUCUUCUGCAGGCUGCU UACGGUUUCGUCCGUGUUGCAGCCGAUCAUCAGCACAUCUAGGUUU CGUCCGGGUGUGACCGAAAGGUAAGAUGGAGAGCCUUGUCCCUGGU UUCAACGAGAA
SL6 [b]	302-343	46	ggCACGUCCAACUCAGUUUGCCUGUUUUACAGGUUCGCGACGUGcc
P2 [b]	726-756	33	ggAUGAAGAUUUUCAAGAAAACUGGAACACUAA
SL1_ext^{SL1} [a]	7-44	38	GGUUUAUACCUUCCCAGGUAACAAACCAACCAACUUUC
SL1_ext^{SL4} [a]	7-33 / 129-148	47	GGUUUAUACCUUCCCAGGUAACAAACCAUAAUUAAUAACUAAUUACU
SL4_ext^{SL1} [a]	86-125 / 34-44	51	gUGUGGCUGUCACUCGGCUGCAUGCUUAGUGCACUCACGCAACCAAC UUUC
P3 [b]	20668-20715	48	GCGUGGCAACCGGGUGUUGCUAUGCCUAAUCUUUACAAAAUGCAAAG A
P3_A [b]	20668-20692	25	GCGUGGCAACCGGGUGUUGCUAUGC
P3_B [b]	20691-20715	25	GCCUAAUCUUUACAAAAUGCAAAGA
ss19T	-	19	GGCACAAUAUAACGUCGCC
ss19B	-	19	GGCGACGUUAUAUUGUGCC
ds19	-	2x19	Annealed ss19T and ss19B
SL_AUA	-	20	GGCGCGCAUAUAAGCGCGCC

[a] 5’UTR element. [b] CDS element.

Supplementary Table 2, related to Table 2:

Supplementary Table 2: SAXS data acquisition, sample details and data analysis for NTD, RNAs alone and their complexes in standard buffer conditions. Values in brackets indicate error ranges. See the equivalent overview for samples in HEPES buffer in the Source Data file ('SI Fig. 8f)'.

**Sample details**	NTD	SL2+3	SL4	SL4ext	Ext	NTD:SL2+3	NTD:SL4	NTD:SL4ext	NTD:Ext
SASBDB accession codes	SASDPK6	SASDPL6	SASDPM6	SASDPN6	SASDPP6	SASDPQ6	SASDPT6	SASDPR6	SASDPS6
Organism	SARS-CoV-2								
Uniprot ID	P0DTC9								
Amino acid range	(G*) 44-180					(G) 44-180	(G) 44-180	(G) 44-180	(G) 44-180
Genomic boundaries (5' -> 3') (NCBI NC_045512.2)		(gg) 40-80	(gg) 86-125 (cc)	(gg) 83-149	(gg) 129-148	(gg) 40-80	(gg) 86-125 (cc)	(gg) 83-149	(gg) 129-148
Ext. coefficient ε 280nm (L mol^{-1} cm^{-1})	26930								
Ext. coefficient ε 260nm (L mol^{-1} cm^{-1})		424500	424500	677800	239800				
Partial specific volume (cm^3 g^{-1})	0.7415	0.5688	0.5684	0.5689	0.5706				
**Data collection parameters**									
Instrument	EMBL P12 BioSAXS beam line, DESY, Hamburg								
Data collection mode	SEC-SAXS								
X-ray wavelength (nm)	0.124								
Energy (keV)	10								
Sample-to-detector distance (m)	3								
Detector	Pilatus 6M								
q measurement range ($Å^{-1}$)	0.0002-0.74								
Scattering intensity scale	Absolute scale, cm^{-1}								
Absolute scaling method	Calibrated to water								
Basis for normalization	Normalized to the intensity of the transmitted beam								
SEC column	Superdex75 Increase 10/300GL (GE Healthcare)								
SEC flow rate (mL min^{-1})	0.7	0.7	0.7	0.7	0.7	0.7	0.7	0.7	0.7
SEC-SAXS buffer	25 mM potassium phosphate, 150 mM potassium chloride, 2 mM TCEP								
SEC temperature (°C)	20								
Sample injection volume (μL)	90								
Protein sample conc. (mg mL^{-1})	4.5					4.5	4.5	4.5	4.5
RNA sample conc. (mg mL^{-1})		3.1	4.3	6.2	2.2	3.1	4.3	6.2	2.2
Exposure time per data frame (s)	0.995								
No. of frames collected	2100	2160	2520	2160	2160	2100	2400	2100	2100
**Data processing**									
SEC-SAXS primary data processing	P12 SASFLOW pipeline and CHROMIXS								

# buffer frames used for averaging	27	238	168	219	119	495	443	66	98
#sample frames used for averaging (frame selection)	13 (1210-1222)	33 (1125-1157)	5 (1363-1367)	9 (1112-1120)	28 (1270-1297)	28 (821-848)	18 (1191-1208)	14 (964-977)	18 (1102-1119)
q working range (Å^{-1})	0.0126-0.60	0.0077-0.68	0.0069-0.62	0.0069-0.49	0.0074-0.65	0.0110-0.65	0.0096-0.65	0.0093- 0.5	0.0082-0.67
Data analysis	PRIMUS (ATSAS 3.1.1) and BioXTAS RAW (2.1.0)								
**Structural parameters**									
**Guinier analysis**									
Data analysis software	PRIMUS (ATSAS 3.1.1)								
Guinier *I*(0)**	0.008258	0.01023	0.04073	0.03701	0.009455	0.02040	0.03408	0.04222	0.01202
R_g, σ (nm)	1.62 (0.009)	2.18 (0.008)	2.02 (0.005)	2.78 (0.007)	1.53 (0.005)	2.40 (0.005)	2.14 (0.003)	3.41 (0.009)	2.14 (0.009)
qR_g limits (point range)	0.21-1.30 (38-280)	0.17-1.30 (20-207)	0.14-1.3 (17-224)	0.19-1.29 (17-160)	0.11-1.30 (19-299)	0.26-1.29 (32-185)	0.21-1.29 (27-210)	0.32-1.30 (26-129)	0.18-1.30 (22-210)
Quality-of-fit parameter (R^2 linear correlation coefficient)	0.972	0.987	0.996	0.995	0.988	0.997	0.998	0.997	0.987
**p(r) analysis**									
Data analysis software	GNOM v5								
I(0) **	0.008265	0.01027	0.04098	0.03739	0.009479	0.02051	0.03429	0.04276	0.01210
R_g, σ (nm)	1.642 (0.012)	2.237 (0.011)	2.088 (0.004)	2.925 (0.009)	1.552 (0.005)	2.464 (0.007)	2.217 (0.004)	3.603 (0.011)	2.224 (0.012)
D_{max} (nm)	6.1	8.0	7.1	10.2	5.4	9.0	7.8	13.5	8.5
Quality of fit (χ^2)	0.945	1.016	0.971	1.078	1.000	0.964	1.034	1.048	0.970
CorMap P-value	0.991	0.766	0.167	0.030	0.352	0.012	0.006	0.024	0.498
Porod volume (nm^3)	23.4	24.1	21.6	32.9	12.6	37.5	24.9	50.6	32.3
**Molecular weight**									
Calculated MW from sequence (kDa)	14.9	13.7	14.1	22.1	7.1	28.6	29.0	37.0	22.0
MW from SAXS data (credibility range) (kDa)	13.5 (12.4-14.5)	14.2 (13.1-15.2)	14.8 (14.5-15.8)	27.6 (25.3-29.3)	8.5 (6.7-9.1)	23.1 (22.1-24.7)	18.1 (17.8-19.0)	36.1 (34.2-38.1)	17.4 (16.5-18.4)
**Atomistic modeling**									
Structure / Model type	X-ray crystal (6M3M)	RNAMasonry	RNAMasonry	RNAMasonry	RNAMasonry				
Data analysis software	CRYSOL (ATSAS 3.1.1)								
q range for fitting (Å^{-1})	0.0132-0.40	0.0076-0.47	0.0068-0.5	0.0068-0.5	0.0074-0.5				
Imposed symmetry	P1								
Quality of fit (χ^2)	0.958	1.189	0.991	1.143	1.280				
CorMap P-value	0.744	0.025	0.102	0.194	0.007				

* Additional artificial N-terminal amino acid from cloning. ** All errors are below 0.2 %.

Supplementary Fig. 1, related to Fig. 1 and Table 1:

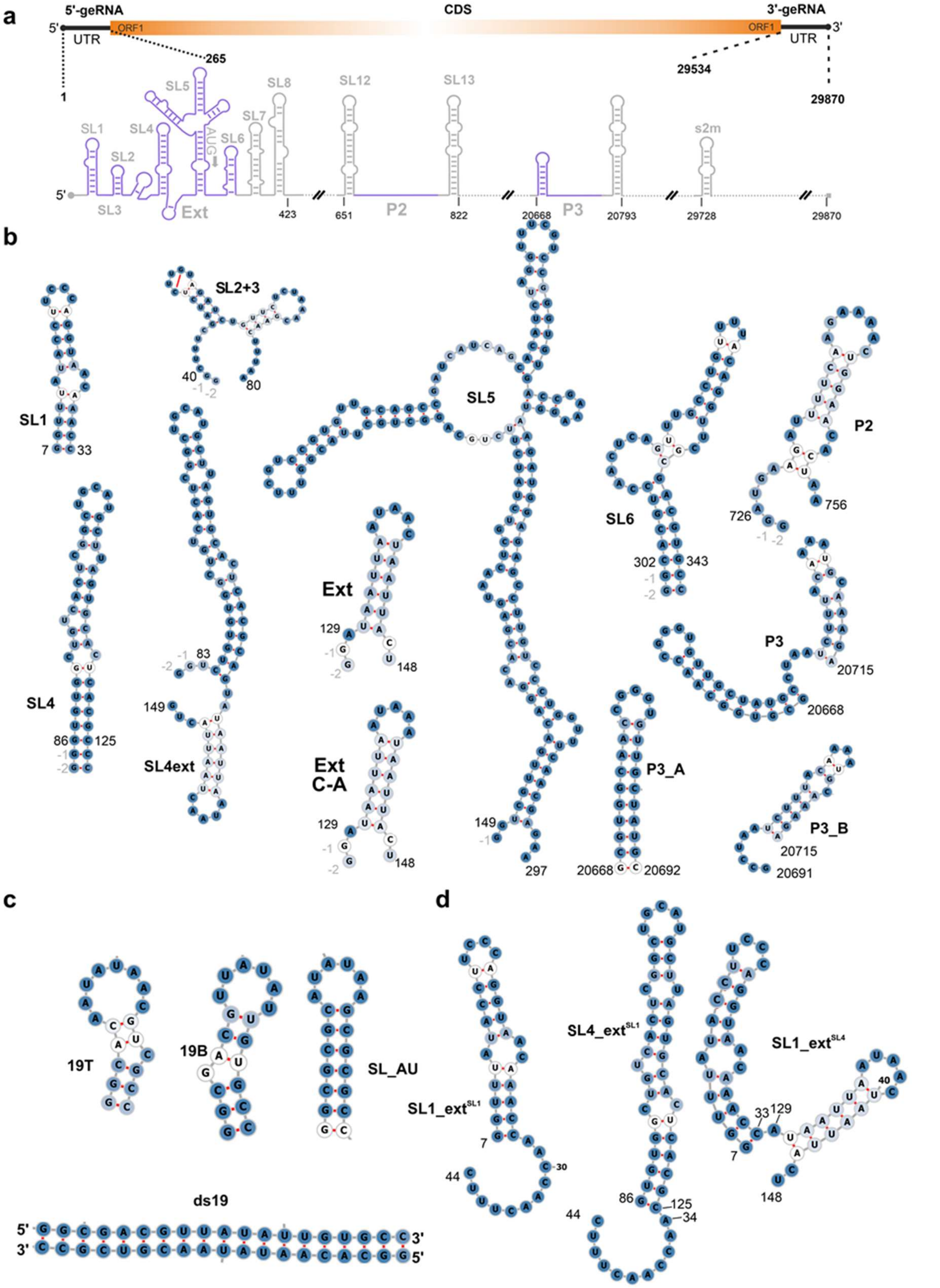

Supplementary Fig. 1 RNA elements used in this study provided with their predicted or experimentally determined folds. **a** Schematic representation of the SCoV-2 geRNA. Indicated above are the boundaries for the 5' genomic end, the coding sequence (CDS) and the 3' genomic end. Highlighted in purple are the elements tested in this study. **b** Vienna RNAfold[22] RNA secondary structure predictions of all viral RNA elements with genomic positions. Note

the additional SL2 loop C-G base-pair in the SL2+3 RNA, which is not predicted but confirmed in multiple studies before[20,23,24]. See also Supplementary Fig. 11 below. **c** Non-viral RNA elements used in this study. **d** Viral geRNA element fusion constructs with nt boundaries. All elements are drawn using the FORNA online tool[25]. Numbers in drawings represent start and end of genomic parts, respectively. Additional nucleotides are non-genomic.

Supplementary Fig. 2, related to Fig. 2:

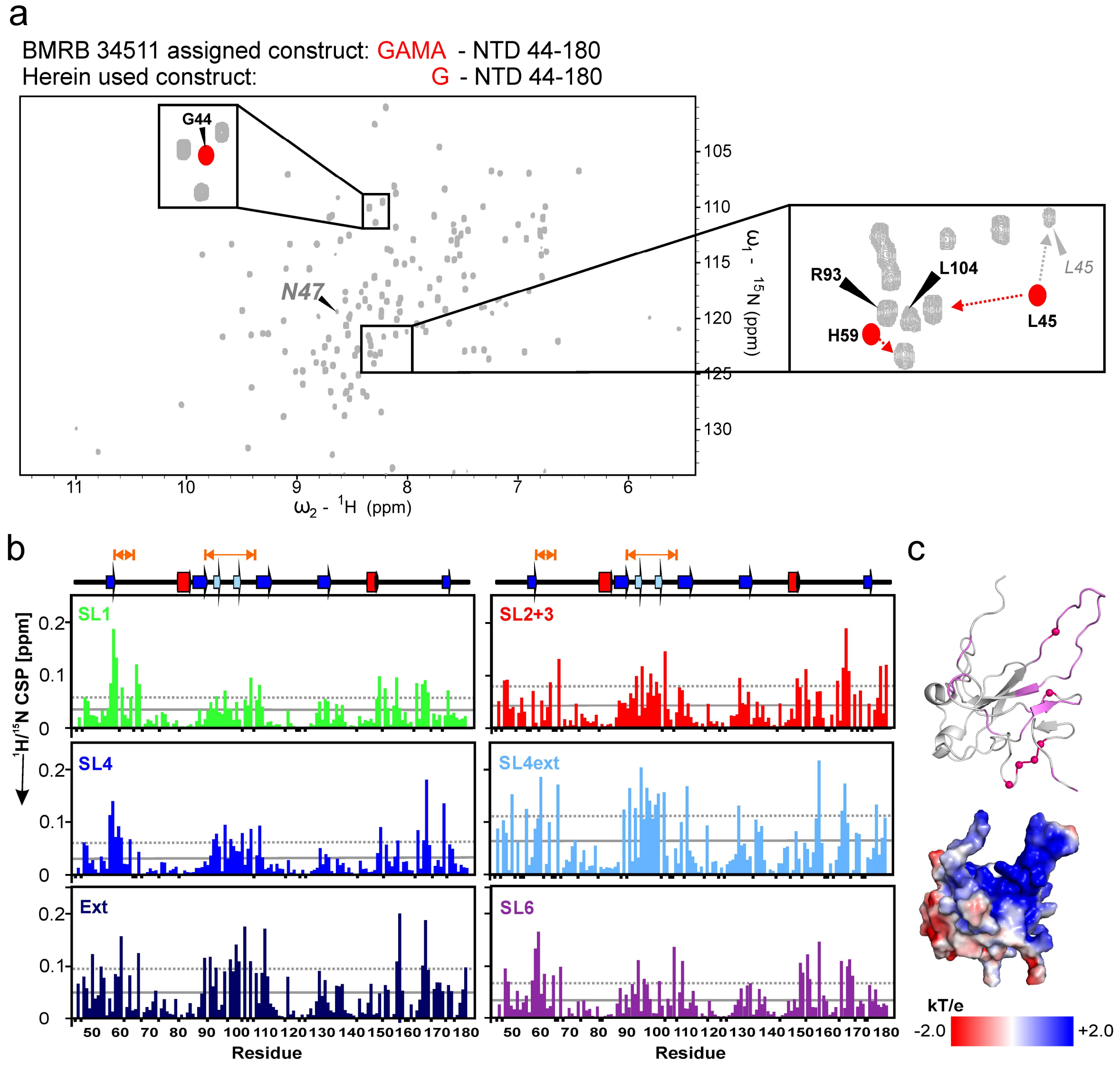

Supplementary Fig. 2 NMR CSP analysis of NTD binding to SCoV-2 5'ge RNA elements. **a** Comparison of the NTD 44-180 (with N-terminal artificial Gly residue from cleavage) in the herein used NTD buffer compared to the BMRB entry 34511[13], highlighting differences of nearby affected residues. Red circles represent NH-resonances as deposited in the BRMB for the NTD 44-180 (including 4 artificial residues GAMA upon cleavage). **b** Combined $^{1}H/^{15}N$ CSPs plotted against the residue number of NTD after addition of 1.2-fold molar excess of SL1, SL2+3, SL4, SL4ext, Ext and SL6, respectively. Lines indicate average CSPs and significance thresholds (average plus SD). NTD secondary structure elements are shown for orientation on top. Negative values represent residues which could not be unambiguously assigned in at least one of the spectra. CSPs upon addition of SL5 could not be quantified due to line-broadening caused by the SL5 molecular weight (see Source Data). **c** Top, compulsory graphical mapping of the NTD RNA-binding epitope. Highlighted in magenta are intersected (for all RNAs) regions with significant CSPs; shared residues that are found for all RNAs are additionally included as magenta spheres on the NTD structure. Bottom, electrostatic surface potential of the NTD derived with the Pymol plugin APBS[26] shown in same orientation as above. All data were recorded at 298K. Source data are provided as a Source Data file.

Supplementary Fig. 3, related to Fig. 2:

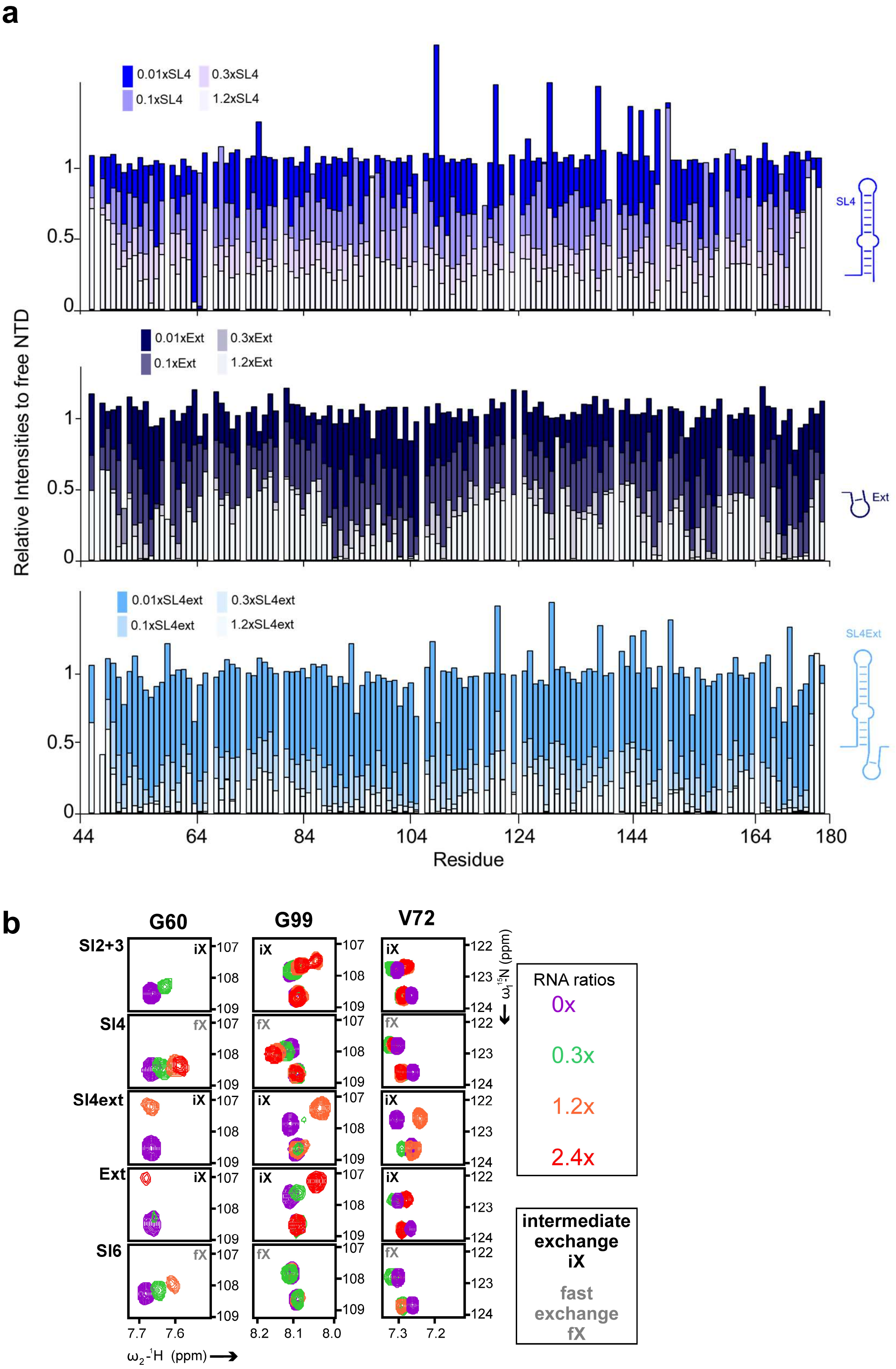

Supplementary Fig. 3 NMR-spectroscopic features of NTD interacting with SCoV-2 viral RNA elements from the 5’ge. **a** Distinct line-broadening patterns indicate different interactions with 5’ge RNA elements. Intensity ratios obtained from titration points of NTD with the given equivalents of SL4, SL4ext and Ext RNAs. Data were measured at 70 µM NTD concentration and, afterwards apo peaks normalized to 100% (not included in plot). RNA titration points are

shown for 0.01x, 0.1x, 0.3x and 1.2x stoichiometries. The juxtapositioning shows that line broadening of NTD resonances upon SL4 titration is equally distributed when compared to titrations with SL4ext and Ext, respectively. Here, pronounced dips in signal intensity can be assigned to the N-terminal stretch (47-66) and the basic finger (90-107). Gaps indicate prolines or residues which could not be unambiguously assigned in at least one of the spectra. **b** Overview of protein NMR-observed exchange regimes between NTD and the 5'ge RNA elements of this study. The zoom-ins show NTD amide groups in apo and RNA-bound forms with ratios as given. For the different RNAs, the spectral comparisons show non-identical exchange regimes as indicated by the respective peak patterns. The type of exchange regime is denoted, and intermediate exchange (iX) regimes indicate dissociation constants of approximately 1-10 µM, while pure fast exchange (fX) is found for constants significantly above 10 µM[27]. All data were recorded at 298 K. Source data are provided as a Source Data file.

Supplementary Fig. 4:

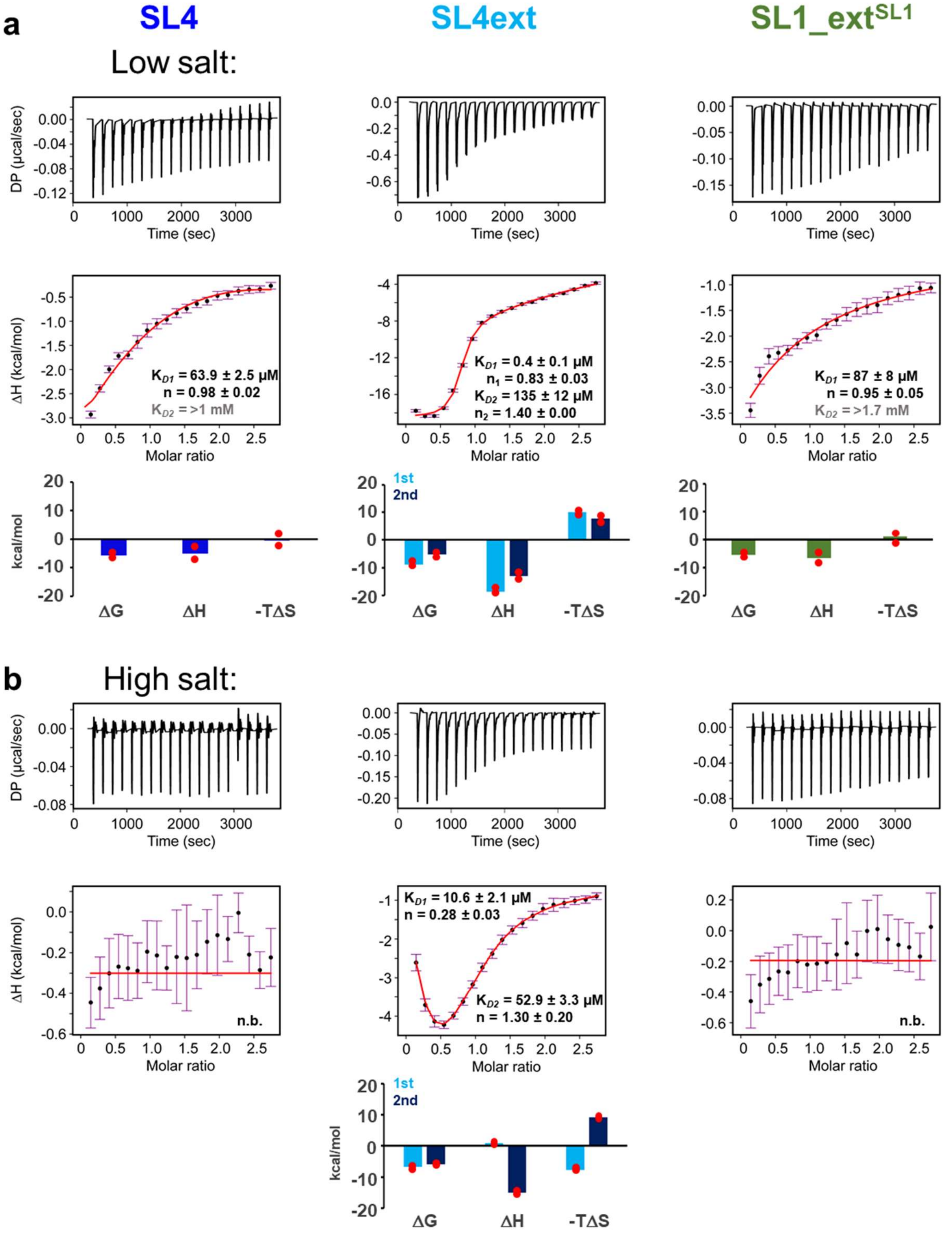

c

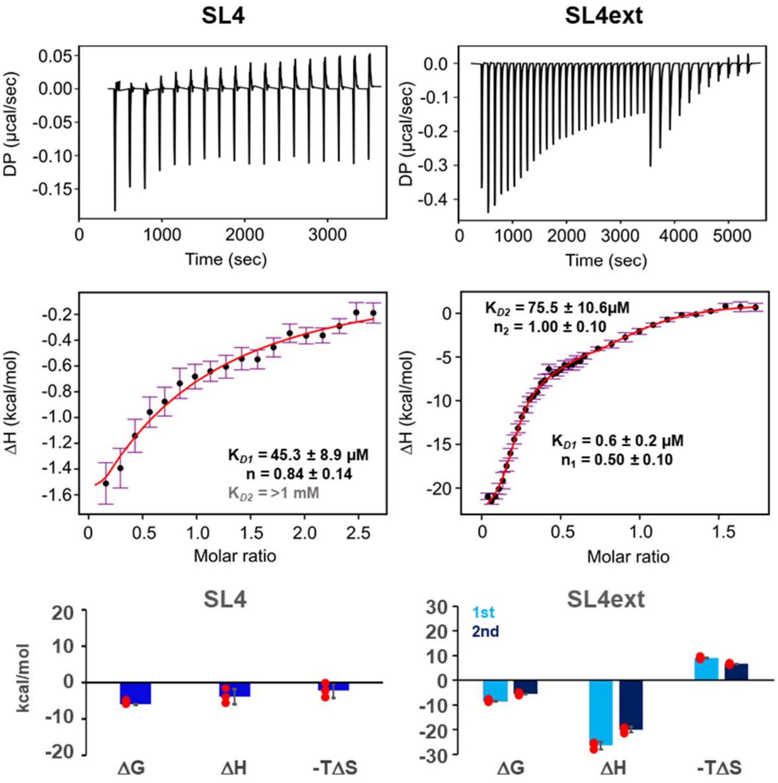

d

	K_D (µM)
SL4	
I)	52.8 ± 12.1
SL4ext	
I)	0.5 ± 0.2
II)	99.3 ± 34.5

Supplementary Fig. 4 ITC. **a** Affinities, stoichiometries and thermodynamic parameters derived from ITC for binding of NTD when titrated to SL4 (left), SL4ext (middle) and the control SL1_ext^{SL1} (right) RNAs at 298 K when measured in low-salt conditions (50 mM KCl). Top, representative thermograms and integrated data fit using a consistent two-step sequential binding model for all RNAs with molar ratios and K_D values as given from duplicates with the absolute error range (n=2 biologically independent samples). For SL4 and SL1_ext^{SL1}, no meaningful thermodynamic parameters were fittable for a second site as indicated by the K_D values >1 mM. Error bars indicate the noise for heat integration at each injection for the representative run and as obtained from the programs NITPIC and SEDPHAT[21]. Below, plot of thermodynamic parameters for the ITC runs given above. Bars are mean values derived from duplicate experiments (red dots). **b** The same as in panel a, but when measured at high-salt conditions (250 mM KCl). No binding was detected for SL4 and SL1_ext^{SL1} RNAs, while SL4ext still provides affinity for the first binding site, which is driven by entropy (in line with NMR titrations at high salt, Fig. 5). The fit also reveals a second, later binding at lower affinity, which is driven by enthalpy. **c** ITC-observed complex formation of RNAs as shown when titrated to NTD provided in the reaction cell at low salt conditions (50 mM KCl). Top, representative thermograms and integrated data fit using a two-step sequential binding model with molar ratios and K_D values as given. Identical to panel (a), no second binding could be fitted to SL4-NTD. Error bars indicate the noise for heat integration at each injection for the representative depicted run and as obtained from the programs NITPIC and SEDPHAT. Bottom, plot of thermodynamic parameters for the ITC runs given above (color code as in panels a and b). Error bars are mean values ± standard deviation obtained from triplicate experiments (absolute values in red dots), i.e. n=3 biologically independent samples. **d** Summary table showing the affinities of NTD binding to SL4 and SL4ext when integrating all ITC data at low salt conditions, i.e. data from panels a and c. Numbers are mean dissociation constants and their overall standard deviations from all replicates of titrations in both directions for the binding events as given (I, andI, II). Find all underlying data of experiments and replicate runs in the Source Data file.

Supplementary Fig. 5, related to Fig. 2:

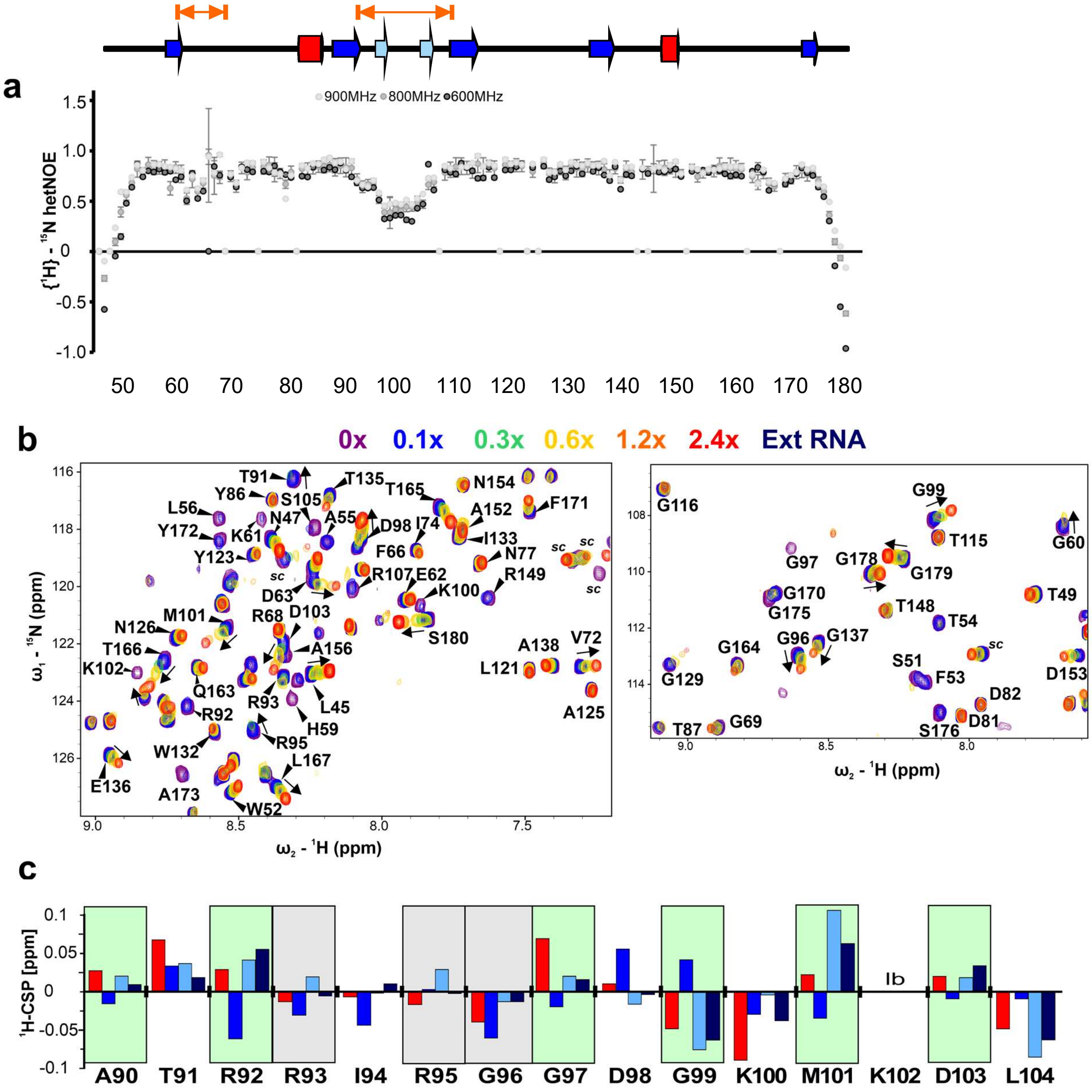

Supplementary Fig. 5 Dynamic residues of the N NTD are utilizable for monitoring CSP patterns related to preferred RNP complex formation. **a** $\{^{1}H\}^{15}N$-hetNOE spectra recorded at proton Larmor frequencies of 600 MHz, 800 MHz and 900 MHz, respectively. Significantly attenuated hetNOE values by magnetic field strength for the basic finger and the N-loop finger indicate a correlated motion, as described for the homologue SCoV NTD[28]. NTD secondary structure elements are shown for orientation on top and arrows indicate the N-loop finger and basic finger, respectively. Errors are derived from the program CCPNMR Analysis[7] and reflect the influence of the spectral signal-to-noise from both underlying spectra. n=1 for each field strength. **b** Two representative zoom-ins of ^{1}H-^{15}N–HSQC overlays showing NTD titrated with Ext RNA at molar ratios depicted above. NTD amide resonance assignments are shown. **c** Absolute ^{1}H-CSPs within the basic finger plotted over residue number. Boxed residues show distinct trajectories upon addition of individual 5'ge RNA elements (grey, individual for each RNA; green, consistent for SL2+3, SL4ext and Ext), K102 line-broadened beyond detection (l.b.). All data were recorded at 298 K.

Supplementary Fig. 6, related to Fig. 2:

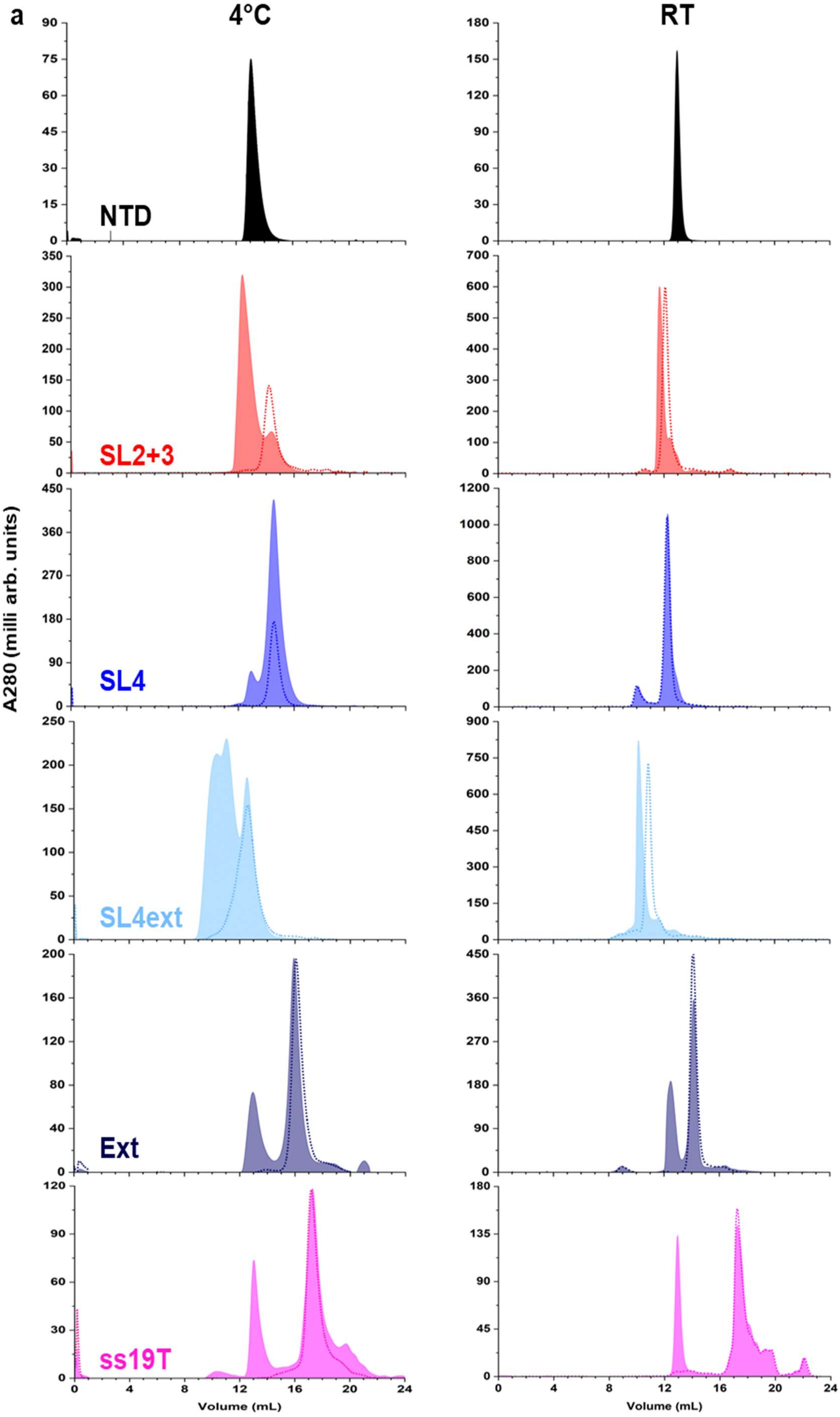

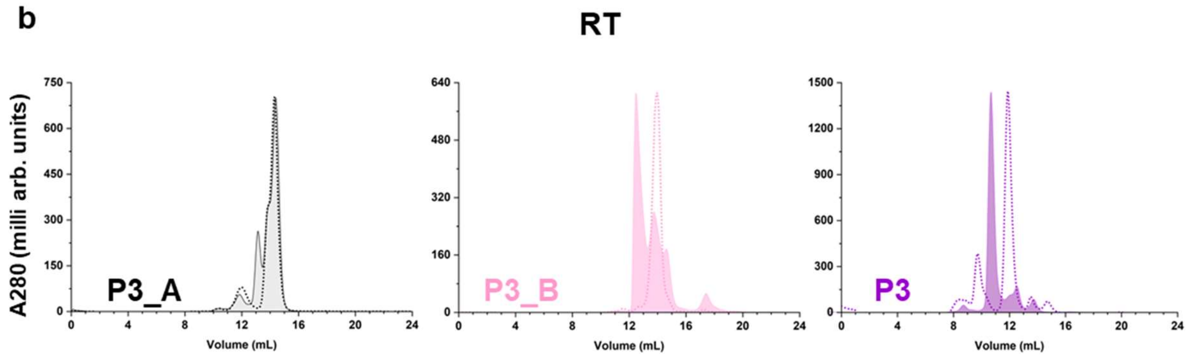

Supplementary Fig. 6. Representative analytical SEC runs showing different stabilities of complexes between NTD and RNAs at two different temperatures. **a** Comparison of analytical SEC runs at 4 °C (left column) and RT (right column) for NTD, selected RNAs of this study as shown (dotted lines, open curves) and their complexes with NTD (filled curves). For the 4 °C runs, RNA amounts differ from those in respective complex runs (i.e., only qualitative readout). For the RT runs, the loaded RNA amount was adjusted to the total RNA amount in runs of complexes with NTD (i.e. yielding a quantitative and qualitative readout for complex formation). Of note, we observed two complex peaks for the SL4ext at low temperature, which likely represents two different conformations of the bound RNA. In accordance with the imino proton assignment spectra at 278K (**Supplementary Fig. 11**), those conformations might represent 1) the completely base-paired SL4 moiety, and 2) the opened-up lower SL4 stem moiety. We do not exclude it may also represent additional NTD molecules bound to RNA in excess stoichiometry, in line with ITC data (Supplementary Fig. 4). **b.** Analytical SEC runs at RT of P3 RNAs as used in this study (dotted lines, open curves) and their complexes with NTD (filled curves). Because different RNA amounts were loaded, RNA-only runs are rescaled to the same maximum as in the complex run for convenience. Find derivable quantifications of these runs in the Source Data file.

Supplementary Fig. 7, related to Fig. 3:

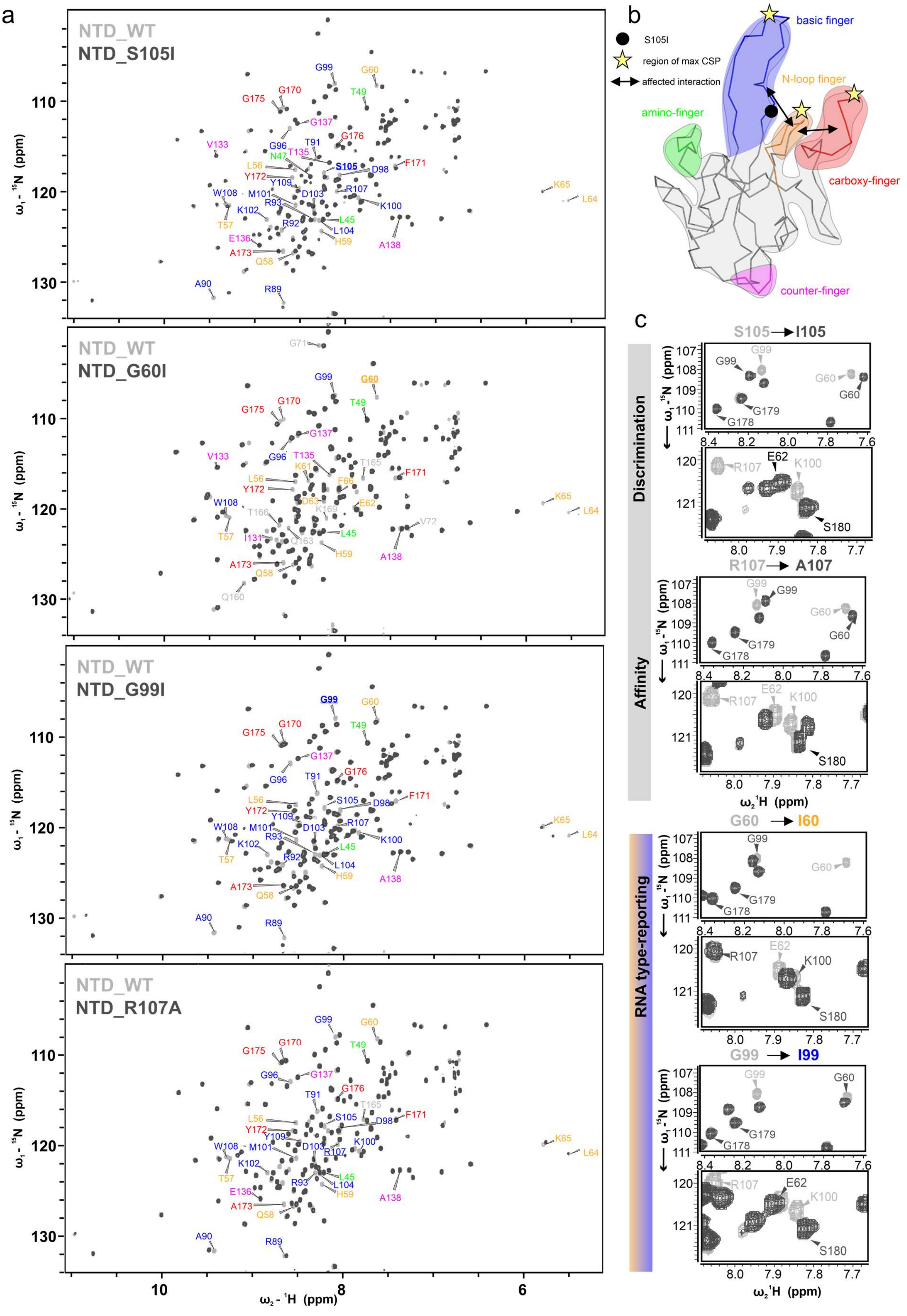

Supplementary Fig. 7 Qualitative analysis of chemical shift modulations caused by single strategic point mutations in the NTD. **a** ^{1}H-^{15}N–HSQC overlay of NTD mutants (dark grey) as indicated in the top left corner of the spectrum vs NTD wildtype (light grey). **b** NTD's hand-like structure with extruding fingers, based on PDB 6YI3[13]. Individual fingers are highlighted in colors. The functional mutation S105I (loss of discrimination) shows a drastic effect on chemical shifts of residues located in the basic finger, the N-loop finger and the carboxy-terminal finger. **c** Zoom-ins of overlays showing a comparison between apo NTD_WT and mutant spectra for selected reporter residues as used in main text **Fig. 3** (upper panel including G99; lower panel including terminal S180). All data were recorded at 298 K. For full spectral views see Source Data.

Supplementary Fig. 8, related to Fig. 4:

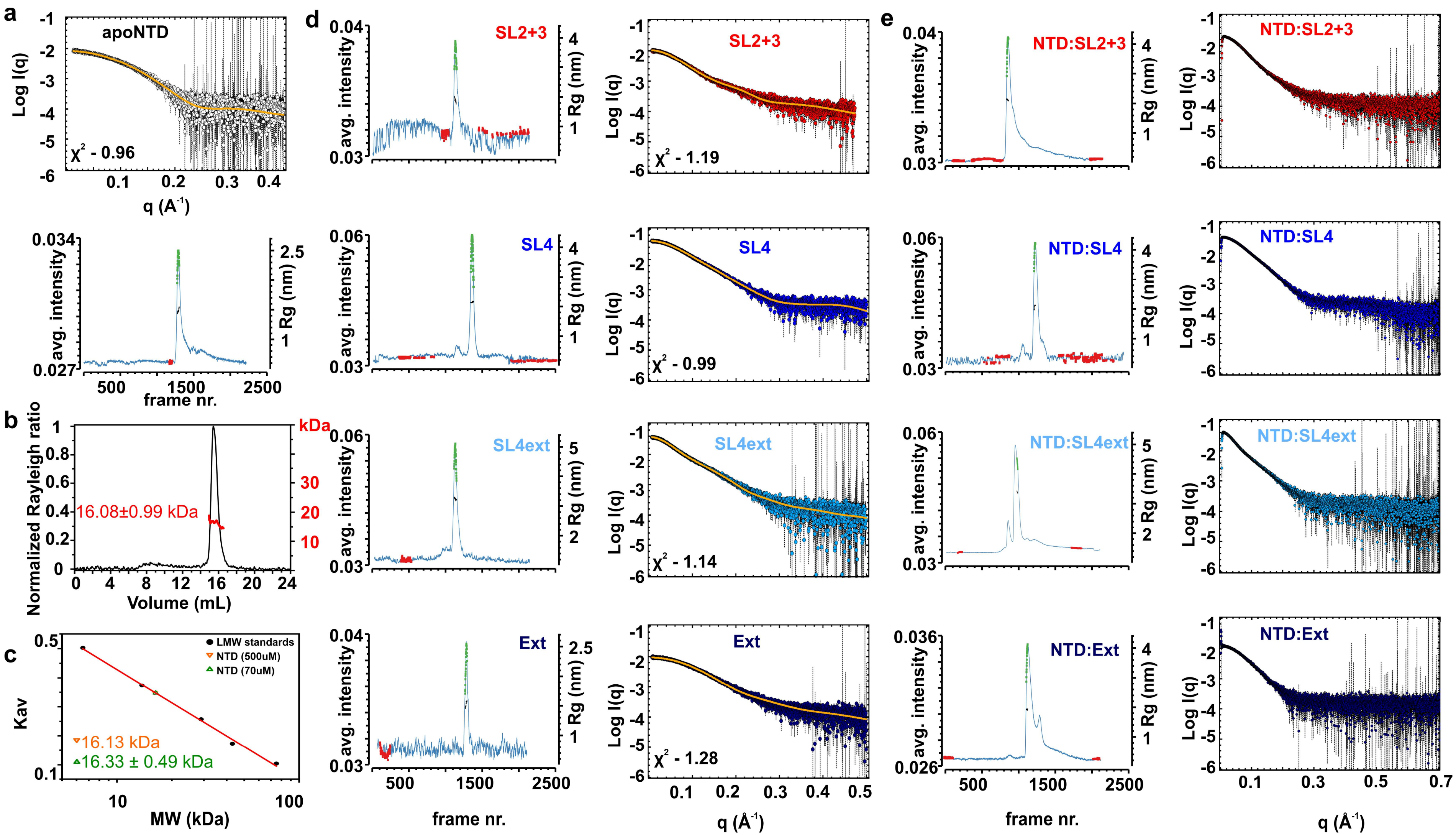

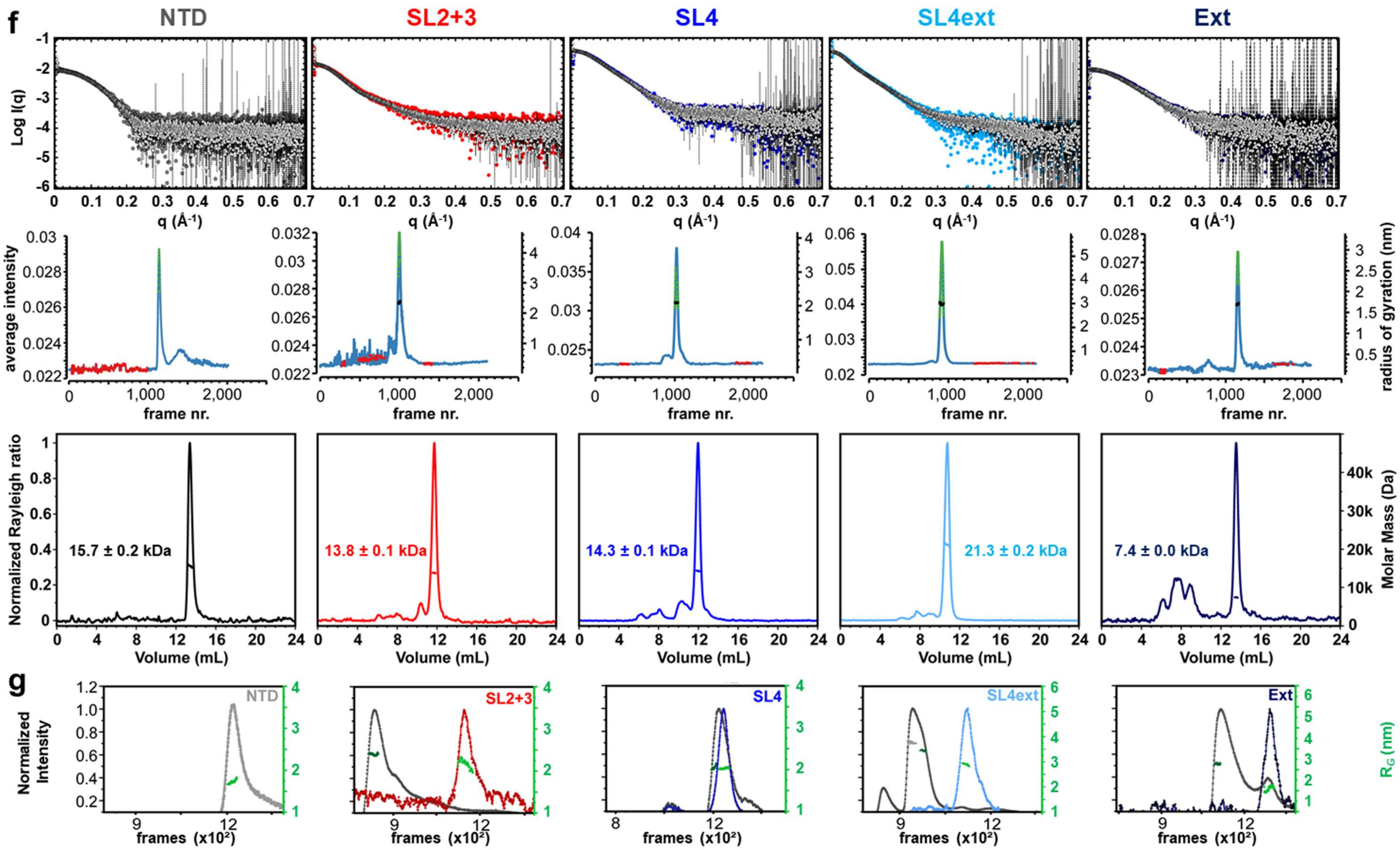

f
NTD
SL2+3
SL4
SL4ext
Ext
Log I(q)
q (Å-1)
average intensity
radius of gyration (nm)
frame nr.
Normalized Rayleigh ratio
Molar Mass (Da)
Volume (mL)
15.7 ± 0.2 kDa
13.8 ± 0.1 kDa
14.3 ± 0.1 kDa
21.3 ± 0.2 kDa
7.4 ± 0.0 kDa
g
Normalized Intensity
frames (x10²)
RG (nm)

Supplementary Fig. 8 SEC-SAXS analysis of monomeric NTD, SCoV-2 5'geRNAs and their complexes. **a** Top, SAXS raw scattering curve of NTD after buffer subtraction (black) overlaid with a calculated scattering curve (orange) derived from PDB entry 6M3M[29]. The fit is given by the χ^2 value. Bottom, SAXS profile of a SEC run for the NTD. The column volume is 24 mL with collected frames as indicated. **b** SEC-MALS analysis of NTD as in parallel with SEC-SAXS. The derived MW across the peak is an average ± SD. **c** Determination of the NTD MW via aSEC and using an MW standard (black dots). Shown is the standard curve of partition coefficient plotted over log MW. The MWs derived for NTD at two concentrations are indicated (n=4 for 70 µM). The expected MW of an NTD monomer, calculated from the amino acid sequence is 14.9 kDa. **d, e** The same as in panel a for RNAs as given, either alone (d, left) or in complex with NTD (e, right). In a, d and e, green frames indicate fractions used for analyses of the sample; red frames show the buffer frame selection (see also Supplementary Table 2). **f** Comparison of SEC-SAXS measurements from individual RNAs and NTD as above in panels d and e to HEPES buffer conditions in order to rule out effects caused from radiation damage. The top row shows an overlay of scattering curves acquired in phosphate (colored, background) with curves derived from samples in HEPES (light grey, front) at identical concentrations and settings. The scattering profiles are very similar as are the SAXS-derived structural parameters. Middle row, depiction of scattering intensity over frames as obtained from SEC-SAXS runs of HEPES samples. Integrated sample frames are shown in green and the buffer frame selection in red. Note that for the NTD alone (left panel) the trace shows the region of buffer frames used to select frames from a quasi-regular spacing (all precise frames are listed in the Source data file). The final, processed scattering profile is used for the overlay in the top row shown above. The bottom panel shows the same SEC run as above, but with the simultaneously monitored MALS trace. The derivable MW is shown as cross section within the main fraction peaks. **g** Proof of complex formation via comparison of increasing R_G. R_G (right axis) is given in individual panels together with relevant traces of X-ray scattering intensity (left axis) recorded over SEC run time (frame number) for NTD alone and comparing RNAs alone (colors) with their NTD-complexes (dark-grey), respectively. Cross-section lines indicate frames as used for downstream analysis in main text Fig. 4 (see Methods for details), plotted by their respective R_G values summarized in main text Table 2. Samples of NTD and RNAs/RNPs in panel g are ordered as in panel f. All SAXS and MALS data were recorded at 293 K or 298 K. Source data are provided as a Source Data file.

Supplementary Fig. 9, related to Fig. 5:

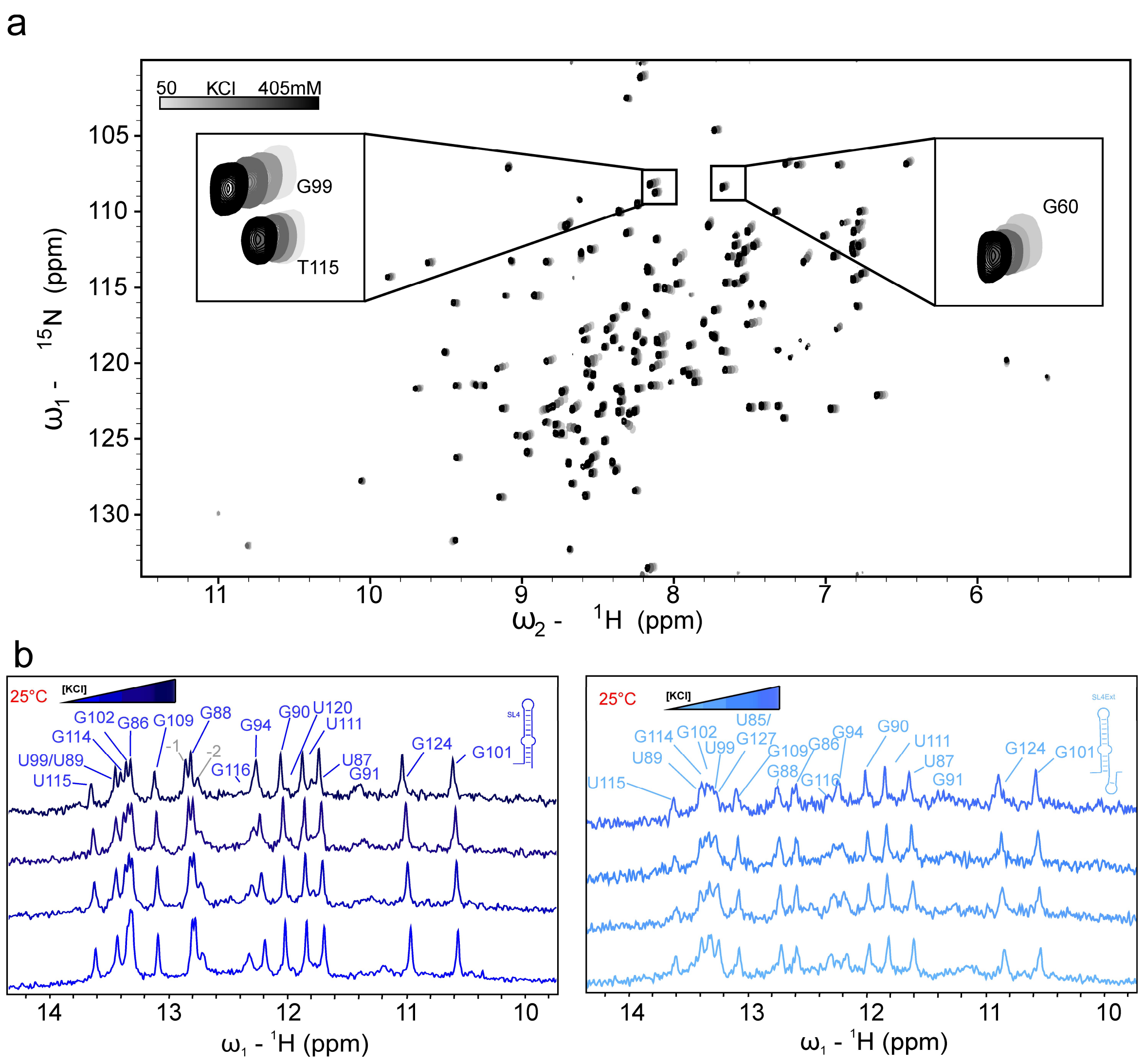

Supplementary Fig. 9 Effect of potassium chloride concentration on NTD and RNAs alone. **a** ^{1}H-^{15}N-HSQC overlays of free NTD (60 µM) with increasing concentrations of salt: 50 mM KCl (light grey), 140 mM KCl (medium grey), 240 mM KCl (dark grey) and 405 mM KCl (black). **b** Imino-^{1}H jump-return spectra of SL4 and SL4ext at increasing salt concentrations. Left, SL4 (40 µM) with 50 mM KCl (blue), 140 mM KCl (red), 240 mM KCl (green) and 405 mM KCl (purple). Right, SL4ext (20 µM) with 50 mM KCl (blue), 140 mM KCl (red), 240 mM KCl (green) and 405 mM KCl (purple). All data were recorded at 298 K. Source data are provided as a Source Data file.

Supplementary Fig. 10, related to Fig. 6:

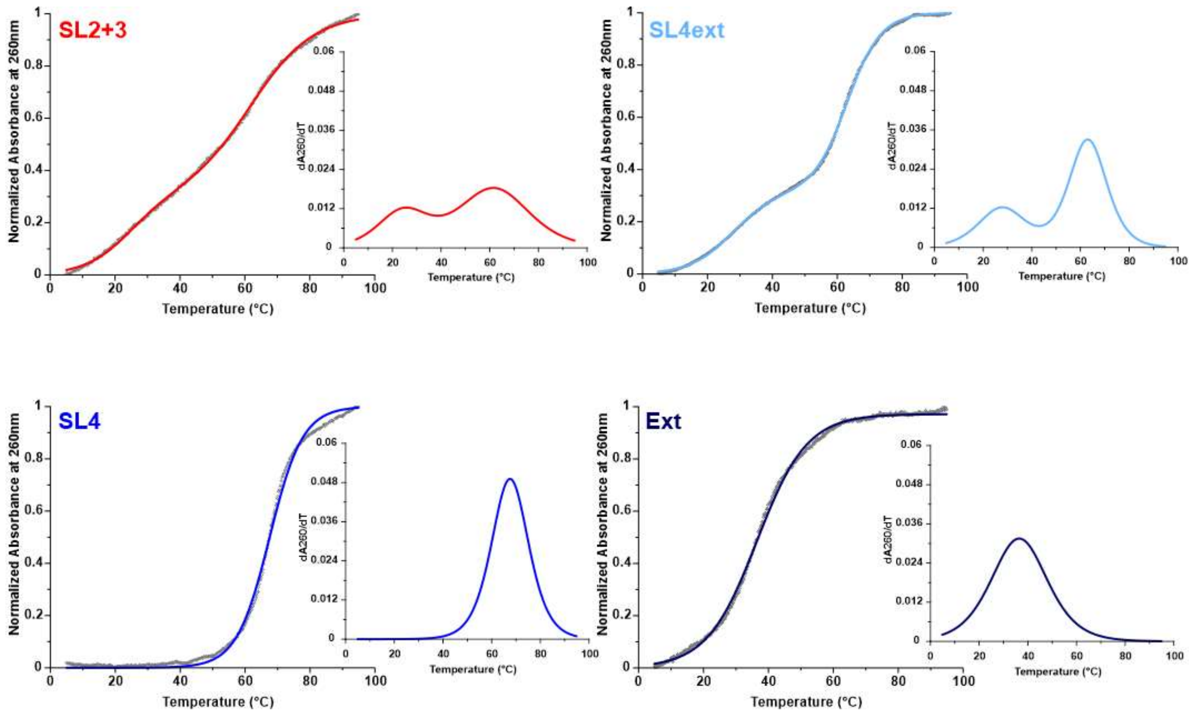

RNA	T_M1 (°C)	Error of fit 1	T_M2 (°C)	Error of fit 2	Red. χ^2	Replicates
SL2+3	24.29	0.23	61.76	0.16	$5.98e^{-5}$	9
SL4	67.56	0.05	-	-	$3.70e^{-4}$	3
SL4ext	27.74	0.12	63.04	0.04	$3.82e^{-5}$	3
Ext	36.46	0.07	-	-	$1.70e^{-4}$	4

Supplementary Fig. 10 Thermal stability of RNA secondary structures in the SL2+3 and SL4-hubs. Top, determination of melting points (T_M) for RNA elements as shown from one representative replicate via normalized absorbance at 260 nm wavelength and raw curve fitting in a CD spectrometer. Curves of the first derivatives as used in main text Fig. 6 are shown as insets. Fit quality is indicated by error of the fit and reduced χ^2 in the table below. The shown T_M values are from the representative replicate. The number of replicates is given. Source data (exact details for all RNAs with n≥3 biologically independent as well as technical replicates) are provided as a Source Data file.

Supplementary Fig. 11, related to Fig. 6+7:

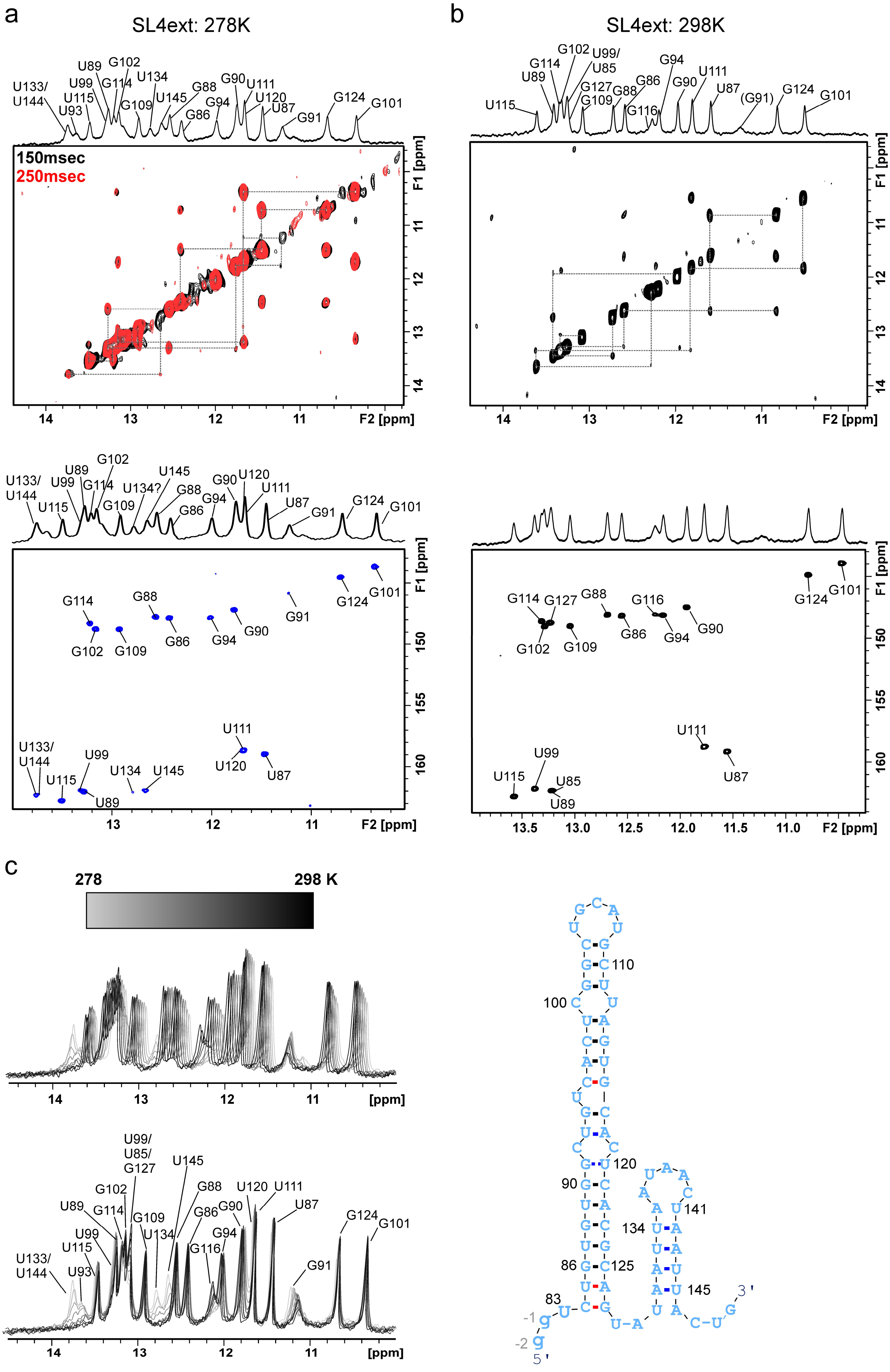

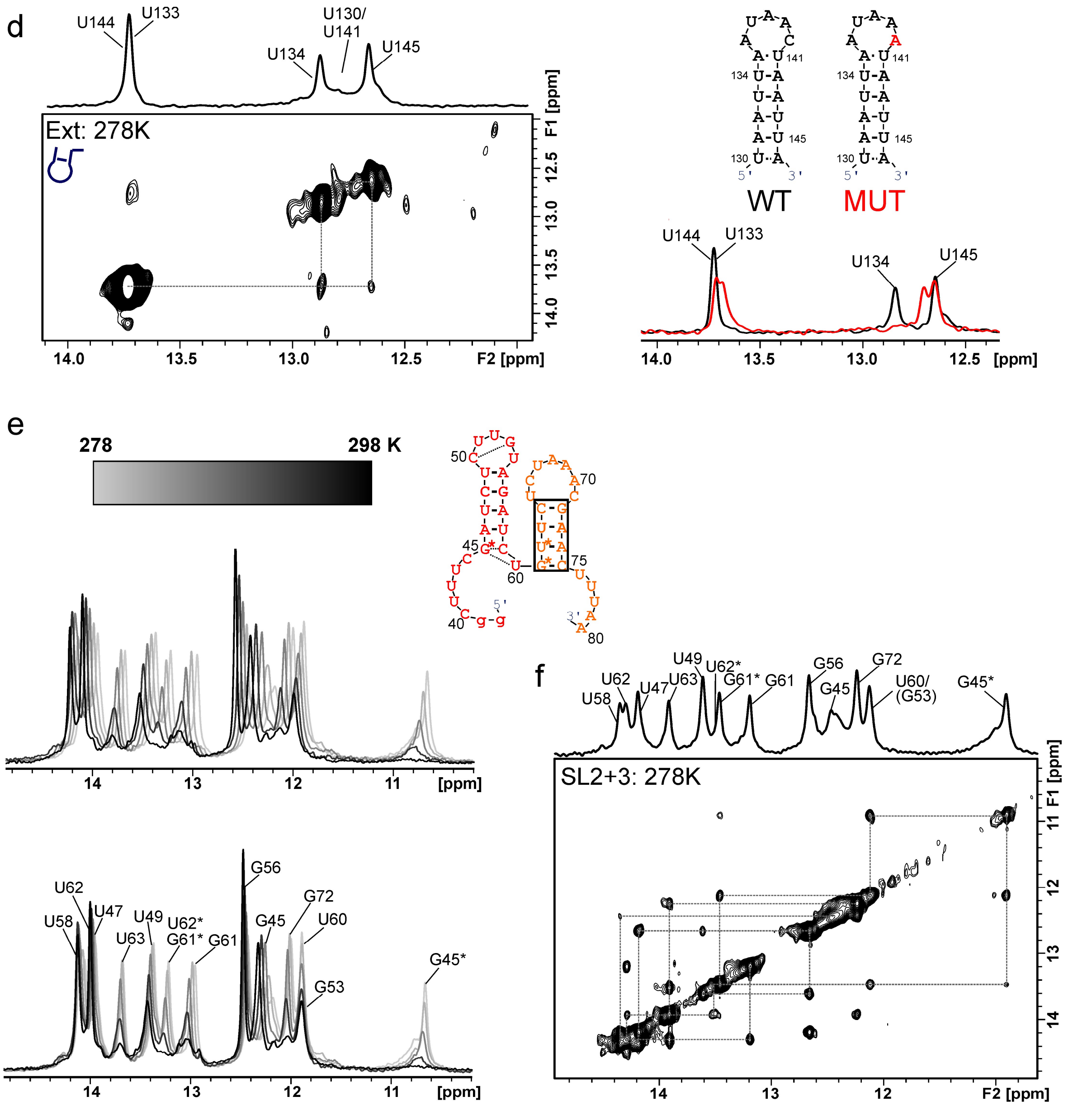

Supplementary Fig. 11 Temperature-dependent imino proton assignments of RNAs preferably bound by NTD. **a** 2D ^{1}H-^{1}H NOESY spectrum of the imino proton region (top) and ^{15}N BEST-TROSY spectrum (bottom) of SL4ext recorded at 278 K. The 1D is shown on top, and assignable resonances are labelled. The NOESY has been recorded at two mixing times, broken lines are included for tracking parts of the sequential walk during the assignment procedure. **b** The same as in panel a, but recorded at 298 K. **c** Temperature titration of SL4ext from 278 K to 298 K. The bottom panel represents the same with a referencing of all spectra to G101. Assignments are included. The scheme on the right depicts the secondary structure of SL4ext. Based on assignments in panels a-c, red bars indicate base-pairs only visible at 298 K, blue bars indicate base-pairs only visible at 278 K. Base-pairs not unambiguously visible or absent are not shown. The scheme indicates that the presence of the folded Ext SL at lower temperature leads to a loss of the most basal base-pairs in SL4, represented by signals for U85 and G127, respectively. This is likely caused by mutual steric hindrance between the two SLs and/or dynamic forces acting on these base-pairs caused by the higher rigidity of the EXT SL conformer. **d** 2D ^{1}H-^{1}H NOESY spectrum of the imino proton region of Ext at 278 K. The 1D is shown on top, and assignable resonances are labelled. The right panel shows an overlay of Ext wildtype and Ext C-A loop-mutant as given by the secondary structure representation.

The mutant induces local CSPs that aid in unambiguously assigning U134 and U145 to the highly symmetric stem-loop. Note that only the SL part of Ext is shown here. **e, f** The same as in panels a and c, but for the SL2+3 RNA. Asterisks indicate a second conformation of the labile SL3 stem loop (the latter only visible at low temperature) based on an alternative base-pairing of SL2 G45 with U60. At the same time, an additional G shows up at 298 K only, likely hidden below U60 at low temperature, which we ascribe to the established SL2 loop G53-C50 base-pair earlier described in references[20,23,24]. Boxed base-pairs are absent at 298 K in line with T_M values shown in main text **Fig. 6** and **Supplementary Fig. 10**. Small letters indicate non-genomic numbering.

Supplementary Fig. 12, related to Fig. 7:

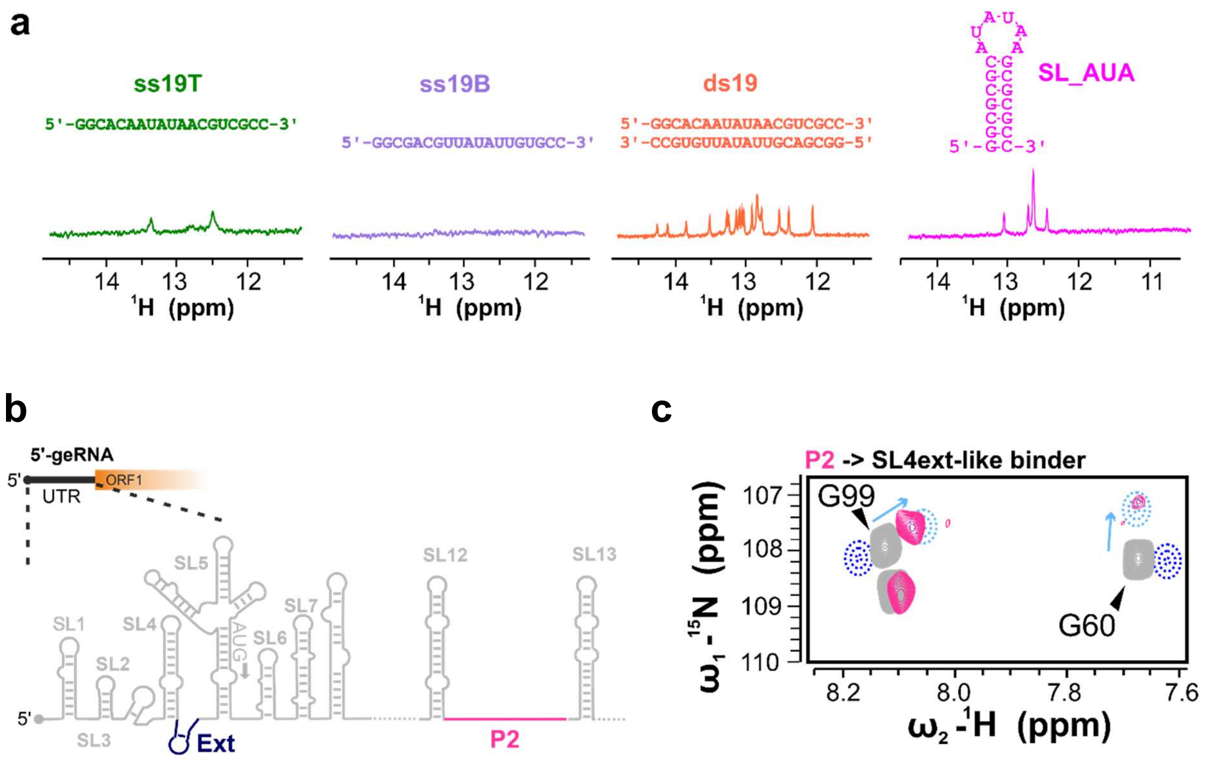

Supplementary Fig. 12 Binding behavior of the SCoV-2 N NTD to non-viral RNAs, the role of RNA sequence, strandedness and fold. **a** ^{1}H-1D spectra of non-viral RNAs used in this study. **b** Schematic depiction of genomic SARS-CoV-2 RNA (nt 1-822). Two principal N-protein binding sites (as predicted by SHAPE coupled to mutational profiling)[30] are indicated and highlighted with respective colors. **c** Spectral excerpt of ^{1}H-^{15}N-HSQC spectrum overlaying free NTD (grey) with viral RNA element P2, including reporters G99 and G60. For comparison of CSP signatures, chemical shifts of 1.2x SL4 (dotted blue lines) and SL4ext (dotted light blue lines) are schematized, respectively. All data were recorded at 298 K.

Supplementary Fig. 13:

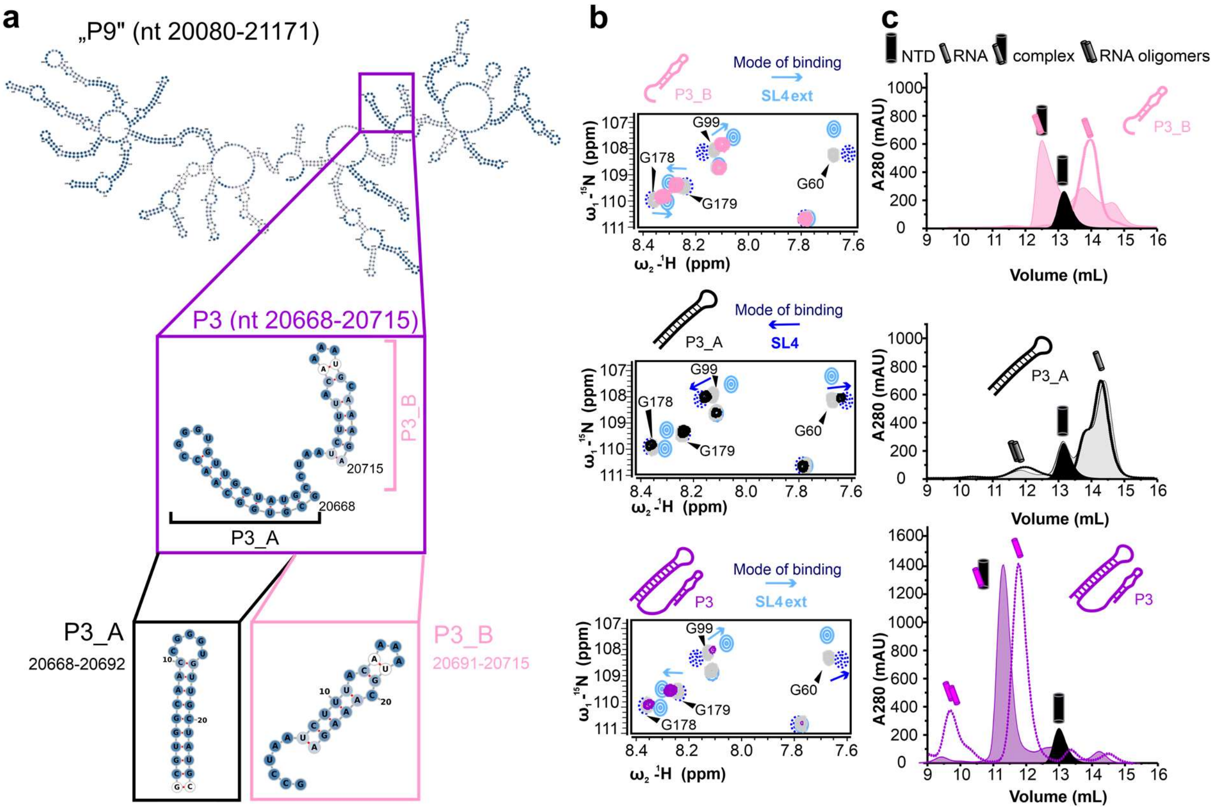

Supplementary Fig. 13 NTD-binding preferences for viral geRNA harbors predictive power towards stably engaged genomic RNA regions. **a** Top, depiction of secondary structure for the packaging-relevant P9 RNA region as described by Syed et al.[31] The zoom-in shows the predicted target site of the SARS-CoV-2 NTD (here termed P3) based on the combination of a stable SL (P3_A) and a labile RNA element (P3_B). Nucleotides are given with respect to the genomic numbering and RNAs shown with folds as suggested by Vienna RNAfold[22] and drawn using the online tool FORNA[25]. **b** Zoom-in of overlays from ^{1}H-^{15}N- HSQC spectra of NTD (grey) with P3 RNAs as from top to bottom: 1.2x molar excess of P3_B (pink), P3_A (black) and P3 (violet), respectively. Within spectral overlays schematic chemical shifts of corresponding 1.2x SL4 (dotted blue lines) and SL4ext (light blue lines) are indicated for comparison of shift modes. Categorization of 'Mode of binding' is indicated above. **c** Corresponding aSEC profiles at RT of complexes of NTD with P3 RNAs as in panel b. Shown are free RNAs (dotted lines, open curves) and their complexes with NTD (coloured filled curves). Free NTD is shown for orientation as black filled curve. All NMR data were recorded at 298 K. Source data are provided as a Source Data file.

Supplementary Fig. 14:

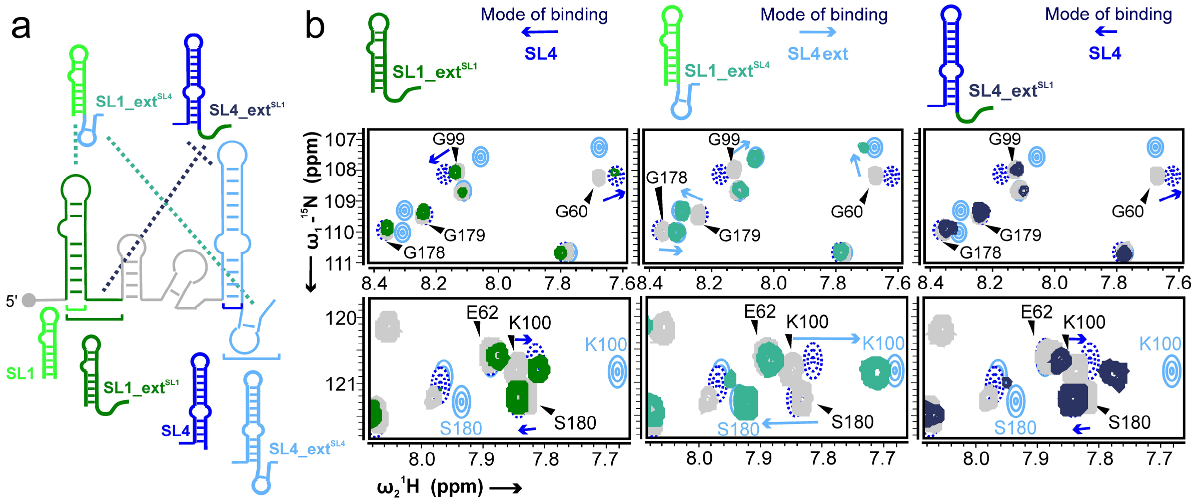

Supplementary Fig. 14 RNA motif swapping shows the N NTD selectively engages with specific sequence extensions (ext) positioned after SLs, but not with arbitrary ones within SCoV-2 viral geRNA. **a** Schematic summary of all tested SL_ext constructs from the viral geRNA 5'UTR: SL1 with the natural subsequent 11 nt extension (SL1_ext^{SL1}), SL4 with the natural subsequent 24 nt extension (SL4_ext^{SL4}, representing the SL4ext construct used in this work) and the swapped versions SL1 with 24 nt extension of SL4 (SL1_ext^{SL4}) and SL4 with the 11 nt extension of SL1 (SL4_ext^{SL1}). **b** Two zoom-ins from overlays (upper panel including G99; lower panel including terminal S180) of ^{1}H-^{15}N- HSQC spectra of NTD (grey) with RNAs from left to right: 1.2x molar excess of SL1_ext^{SL1} (green), SL1_ext^{SL4} (turquois) and SL4_ext^{SL1} (dark blue), respectively. Within spectral overlays schematic chemical shifts of corresponding 1.2x SL4 (dotted blue lines) and SL4ext (in this context referred to as SL4_ext^{SL4}, light blue lines) are indicated for comparison of CSP signatures, i.e., the type of binding. The respective category of binding mode is indicated above. All data were recorded at 298 K. Source data are provided as a Source Data file.

Supplementary Fig. 15:

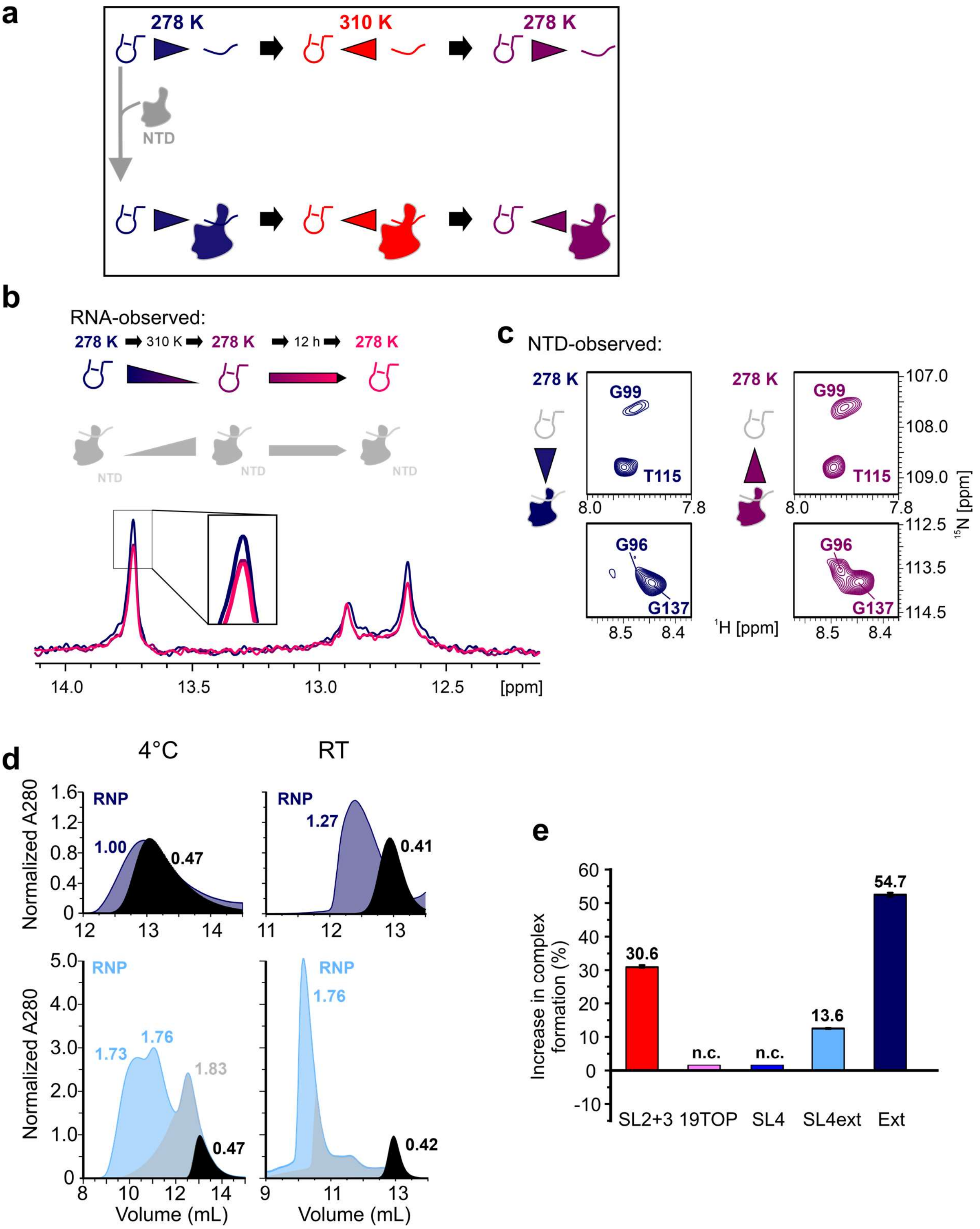

Supplementary Fig. 15 The NTD modulates equilibria of target RNA foldamers towards ssRNA moieties. **a** Coronaviral nucleocapsids were suggested to function as chaperones, i.e., actively unfolding/refolding target RNAs as a central function[32]. Model and experimental working hypothesis for interacting with unfolded conformers of target RNAs. A temperature rise increases accessibility to the binding-competent foldamers of RNAs, which remain tightly bound to NTD independent of subsequent temperature lowering. **b** Verification of model in c as observed through folded Ext RNA NMR resonances acquired at 278 K. Spectra of the identical complex sample with NTD have been recorded while undergoing the temperature

route as given in a. **c** Verification of model in panel a as observed through protein-detectable NTD amide NMR resonances at 278 K. Zoom-ins show representative peaks depicting the increase in complex formation by the accessibility of more ss Ext RNA after temperature cycling and return to 278 K. **d** Comparison of aSEC runs performed at either 4 °C or RT. The zoom-ins show regions of complex retention volumes for the two targets Ext and SL4ext when run in presence of NTD, and free protein as reference. Absorption at 280 nm is normalized to free NTD protein. The corresponding numbers quoted on the chromatograms are the A260/A280 ratios for denoted peaks (see also **Supplementary Fig. 6 and methods**). Of note, we observed two complex peaks for the SL4ext at low temperature, which likely represents two different conformations of the bound RNA. In accordance with the imino proton assignment spectra at 278K (**Supplementary Fig. 11**), those conformations might represent 1) the completely base-paired SL4 moiety, and 2) the opened-up lower SL4 stem moiety. We also do not exclude it may also represent additional NTD molecules bound to RNA in excess stoichiometry, in line with ITC data (Supplementary Fig. 4). RNP, RNA-protein complexes. **e** Bar plot representing the relative increase in complex formation for the RNAs as given upon temperature increase as taken from panel d. Error bars were estimated from the observed deviations for exactly the same sample when run at two different FPLCs at 4°C and RT, and equipped with two different columns (n=1 each). A general error estimate of reproducibility in peak integration at identical conditions was carried out for NTD and selected RNAs using n=2 biologically independent samples (see Source Data file for details).

Supplementary Fig. 16, related to Fig. 7:

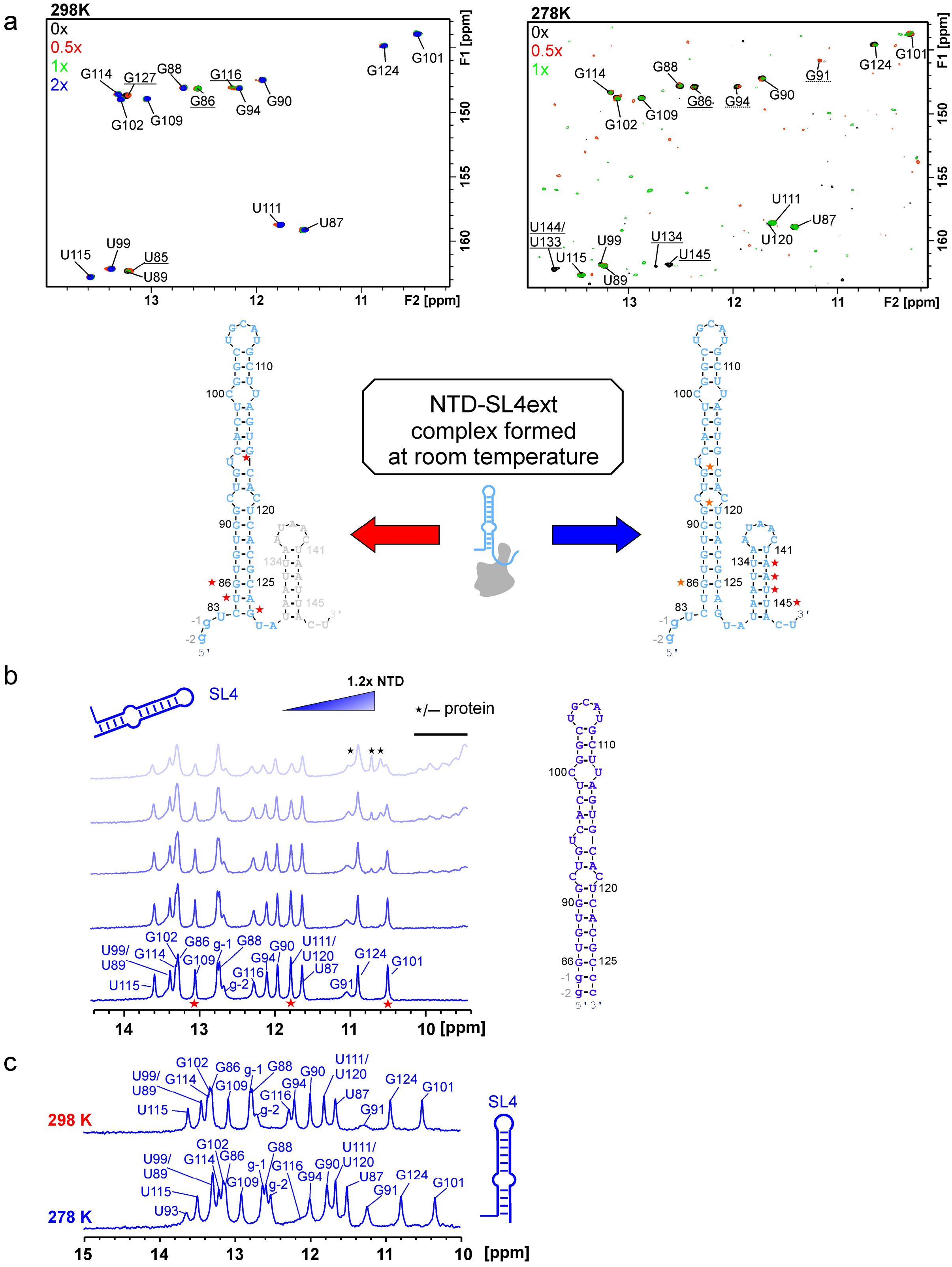

Supplementary Fig. 16 Mapping of NTD-binding sites on SL4 and SL4ext using RNA-observed NMR. **a** Mapping of NTD binding sites to SL4ext when titrated at room temperature and visualized at either 298 K (left) or 278 K (right) to allow tracking Ext residues. ^{15}N BEST-TROSY spectra were recorded for imino correlations at RNA:protein ratios as given. Significantly affected bases are displayed underlined and marked with asterisks in the scheme

below. Note that Ext peaks are invisible at 298 K (grey-shaded), while the binding of NTD to Ext is still sensed in the adjacent SL4 basal region. At 278 K, selective, instant line-broadening of Ext is observed upon addition of NTD. Minor affects are seen for loop regions in SL4, in line with a subordinate binding as also seen in ITC (see Suppl. Fig. 4). Note that general line-broadening after NTD-binding leads to a lower S/N at 278 K. **b** Display of imino-^{1}H-spectra of SL4 alone (bottom) and at increasing stoichiometric ratios of NTD. Unambiguously assignable effects (to particular imino resonances/regions) are indicated with asterisks below the apo spectrum. Despite its stable and robust structure, SL4 imino peaks[19,20] adjacent to bulges or loops are found affected by line-broadening in contrast to those of base paired regions. We conclude that even in the stable SL4 context, the NTD is capable of recognizing regions with ssRNA character, which again emphasizes NTD's ability to precisely differentiate between available RNA motifs. **c** Comparison of imino-^{1}H-spectra recorded at either 298 K as in panel b or at 278 K with assignments as published before[19]. The assignment at low temperature is based on an assignment at 283 K shown in[20]. Small letters indicate non-genomic numbering.

Supplementary References

1 Blanchet, C. E. *et al.* Versatile sample environments and automation for biological solution X-ray scattering experiments at the P12 beamline (PETRA III, DESY). *J Appl Crystallogr* **48**, 431-443, doi:10.1107/S160057671500254X (2015).

2 Franke, D., Kikhney, A. G. & Svergun, D. I. Automated acquisition and analysis of small angle X-ray scattering data. *Nuc. Inst. Meth. A.* **689**, 52-59, doi:doi.org/10.1016/j.nima.2012.06.008 (2012).

3 Panjkovich, A. & Svergun, D. I. CHROMIXS: automatic and interactive analysis of chromatography-coupled small-angle X-ray scattering data. *Bioinformatics* **34**, 1944-1946, doi:10.1093/bioinformatics/btx846 (2018).

4 Manalastas-Cantos, K. *et al.* ATSAS 3.0: expanded functionality and new tools for small-angle scattering data analysis. *J Appl Crystallogr* **54**, 343-355, doi:10.1107/S1600576720013412 (2021).

5 Svergun, D. I. Determination of the regularization parameter in indirect-transform methods using perceptual criteria. *J. Appl. Crystallogr.* **25**, 495-503, doi:10.1107/S0021889892001663 (1992).

6 Hopkins, J. B., Gillilan, R. E. & Skou, S. BioXTAS RAW: improvements to a free open-source program for small-angle X-ray scattering data reduction and analysis. *J Appl Crystallogr* **50**, 1545-1553, doi:10.1107/S1600576717011438 (2017).

7 Hajizadeh, N. R., Franke, D., Jeffries, C. M. & Svergun, D. I. Consensus Bayesian assessment of protein molecular mass from solution X-ray scattering data. *Sci Rep* **8**, 7204, doi:10.1038/s41598-018-25355-2 (2018).

8 Trewhella, J. *et al.* 2017 publication guidelines for structural modelling of small-angle scattering data from biomolecules in solution: an update. *Acta Crystallogr D Struct Biol* **73**, 710-728, doi:10.1107/S2059798317011597 (2017).

9 Chojnowski, G. Z., R.; Magnus, M.; Bujnicki, J.M. RNA fragment assembly with experimental restraints. *bioRxiv* **Preprint**, doi:10.1101/2021.02.08.430198 (2021).

10 Svergun, D. B., C.; Koch, M. H. J. CRYSOL - a Program to Evaluate X-ray Solution Scattering of Biological Macromolecules from Atomic Coordinates. *J. App. Cryst.* **28**, 768-773, doi:10.1107/S0021889895007047 (1995).

11 Valentini, E., Kikhney, A. G., Previtali, G., Jeffries, C. M. & Svergun, D. I. SASBDB, a repository for biological small-angle scattering data. *Nucleic Acids Res* **43**, D357-363, doi:10.1093/nar/gku1047 (2015).

12 Graewert, M. A. *et al.* Adding Size Exclusion Chromatography (SEC) and Light Scattering (LS) Devices to Obtain High-Quality Small Angle X-Ray Scattering (SAXS) Data. *Crystals* **10**, 975 (2020).

13 Dinesh, D. C. *et al.* Structural basis of RNA recognition by the SARS-CoV-2 nucleocapsid phosphoprotein. *PLoS Pathog.* **16**, e1009100 (2020).

14 Redzic, J. S. *et al.* The Inherent Dynamics and Interaction Sites of the SARS-CoV-2 Nucleocapsid N-Terminal Region. *J Mol Biol* **433**, 167108, doi:10.1016/j.jmb.2021.167108 (2021).

15 Clubb, R. T., Thanabal, V. & Wagner, G. A new 3D HN(CA)HA experiment for obtaining fingerprint HN-Halpha peaks in 15N- and 13C-labeled proteins. *J Biomol NMR* **2**, 203-210, doi:10.1007/BF01875531 (1992).

16 Schleucher, J., Sattler, M. & Griesinger, C. Coherence Selection by Gradients without Signal Attenuation: Application to the Three-Dimensional HNCO Experiment. *Angew Chem Int Ed Engl* **32**, 1489-1491, doi:doi.org/10.1002/anie.199314891 (1993).

17 Vranken, W. F. *et al.* The CCPN data model for NMR spectroscopy: Development of a software pipeline. *Proteins-Structure Function and Bioinformatics* **59**, 687-696 (2005).

18 Lee, W., Tonelli, M. & Markley, J. L. NMRFAM-SPARKY: enhanced software for biomolecular NMR spectroscopy. *Bioinformatics* **31**, 1325-1327, doi:10.1093/bioinformatics/btu830 (2015).

19 Vogele, J. *et al.* (1)H, (13)C, (15)N and (31)P chemical shift assignment for stem-loop 4 from the 5'-UTR of SARS-CoV-2. *Biomol NMR Assign*, doi:10.1007/s12104-021-10026-7 (2021).

20 Wacker, A. *et al.* Secondary structure determination of conserved SARS-CoV-2 RNA elements by NMR spectroscopy. *Nucleic Acids Res*, doi:10.1093/nar/gkaa1013 (2020).
21 Brautigam, C. A., Zhao, H., Vargas, C., Keller, S. & Schuck, P. Integration and global analysis of isothermal titration calorimetry data for studying macromolecular interactions. *Nat Protoc* **11**, 882-894, doi:10.1038/nprot.2016.044 (2016).
22 Hofacker, I. L. Vienna RNA secondary structure server. *Nucleic Acids Res* **31**, 3429-3431, doi:10.1093/nar/gkg599 (2003).
23 Lee, C. W., Li, L. & Giedroc, D. P. The solution structure of coronaviral stem-loop 2 (SL2) reveals a canonical CUYG tetraloop fold. *FEBS Lett* **585**, 1049-1053, doi:10.1016/j.febslet.2011.03.002 (2011).
24 Bottaro, S., Bussi, G. & Lindorff-Larsen, K. Conformational Ensembles of Noncoding Elements in the SARS-CoV-2 Genome from Molecular Dynamics Simulations. *J Am Chem Soc* **143**, 8333-8343, doi:10.1021/jacs.1c01094 (2021).
25 Kerpedjiev, P., Hammer, S. & Hofacker, I. L. Forna (force-directed RNA): Simple and effective online RNA secondary structure diagrams. *Bioinformatics* **31**, 3377-3379, doi:10.1093/bioinformatics/btv372 (2015).
26 Dolinsky, T. J., Nielsen, J. E., McCammon, J. A. & Baker, N. A. PDB2PQR: an automated pipeline for the setup of Poisson-Boltzmann electrostatics calculations. *Nucleic Acids Res* **32**, W665-667, doi:10.1093/nar/gkh381 (2004).
27 Williamson, M. P. Using chemical shift perturbation to characterise ligand binding. *Prog Nucl Magn Reson Spectrosc* **73**, 1-16, doi:10.1016/j.pnmrs.2013.02.001 (2013).
28 Clarkson, M. W. *et al.* Mesodynamics in the SARS nucleocapsid measured by NMR field cycling. *J Biomol NMR* **45**, 217-225, doi:10.1007/s10858-009-9347-6 (2009).
29 Kang, S. S. *et al.* Crystal structure of SARS-CoV-2 nucleocapsid protein RNA binding domain reveals potential unique drug targeting sites. *Acta Pharmaceutica Sinica B* **10**, 1228-1238, doi:10.1016/j.apsb.2020.04.009 (2020).
30 Iserman, C. *et al.* Genomic RNA Elements Drive Phase Separation of the SARS-CoV-2 Nucleocapsid. *Mol. Cell.* **80**, 1078-1091 (2020).
31 Syed, A. M. *et al.* Rapid assessment of SARS-CoV-2-evolved variants using virus-like particles. *Science* **374**, 1626-1632, doi:10.1126/science.abl6184 (2021).
32 Zuniga, S. *et al.* Coronavirus nucleocapsid protein is an RNA chaperone. *Virology* **357**, 215-227, doi:10.1016/j.virol.2006.07.046 (2007).
